# Ergothioneine-rich water extracts of *Hericium erinaceus* HE-17 alleviate Alzheimer’s disease in mice by regulating oxidative stress, inflammation, and the gut microenvironment

**DOI:** 10.3389/fnut.2026.1835714

**Published:** 2026-05-21

**Authors:** Ping-Ping Gao, Feng-Long Zhou, Guo-Bang Zheng, Ying-Li Liu, Shu-Jie Cheng, Zhi-Wei Ye, Ya-Ting Liang, Le Liu, Li-Qiong Guo, Jun-Fang Lin

**Affiliations:** 1 Department of Bioengineering, College of Food Science, South China Agricultural University, Guangzhou, China; 2 School of Food Science, Guangdong Pharmaceutical University, Zhongshan, China; 3 Key Laboratory of Geriatric Nutrition and Health (Beijing Technology and Business University), Ministry of Education, Beijing Technology and Business University (BTBU), Beijing, China; 4 Department of Food Nutrition and Safety/National R&D Center for Chinese Herbal Medicine Processing, School of Engineering, China Pharmaceutical University, Nanjing, China

**Keywords:** ergothioneine, functional food, gut microbiota, *Hericium erinaceus*, oxidative stress

## Abstract

Alzheimer’s disease (AD) imposes a significant global disease burden, necessitating simple dietary interventions to prevent or delay disease progression. However, the neuroprotective effects of ergothioneine (EGT)-rich natural extracts as dietary supplements remain largely unexplored. This study aimed to screen a high EGT-producing *Hericium erinaceus* strain (HE-17) from 29 isolates, optimize its culture conditions and amino acid supplementation using single-factor and orthogonal experiments, and evaluate the neuroprotective effects of its extract in an APP/PS1 transgenic mouse model. Cognitive function, neuronal damage, inflammation, oxidative stress, and gut microbiota composition were assessed using the Morris Water Maze test, histopathology, immunofluorescence, biochemical assays, enzyme-linked immunosorbent assay (ELISA), and 16S rRNA gene sequencing. The results showed that the water extract of *H. erinaceus* HE-17 (WEH) contained 2.57 ± 0.14 mg/g EGT and exhibited no acute toxicity in mice. High-dose WEH (2 g/kg BW/d, containing 5.76 mg/kg EGT), low-dose WEH (0.50 g/kg BW/d, containing 1.44 mg/kg EGT), and pure EGT (1.44 mg/kg), administered for 90 days, were found to improve cognitive function and enhance spatial learning and memory, while reducing Aβ aggregation and tau phosphorylation. Meanwhile, WEH supplementation was associated with decreased pro-inflammatory cytokine levels (IL-1β, IL-6, and TNF-*α*) in both the brain and serum. It also reduced oxidative stress markers while increasing antioxidant indicators in brain tissue, with the high-dose group showing the most pronounced effects in APP/PS1 mice. Gut microbiota analysis revealed that low-dose WEH was associated with a reduced abundance of *Fusobacteriota* and *Proteobacteria* and an increased abundance of *Lactobacillus*. These findings suggest that WEH may help mitigate AD-related pathological changes in APP/PS1 mice.

## 1 Introduction

Alzheimer’s disease (AD) is a progressive neurodegenerative disorder and a leading cause of dementia and age-related cognitive decline (1). Its key pathological features include *β*-amyloid (Aβ) aggregation and tau hyperphosphorylation. Aβ accumulation induces oxidative stress and neuronal damage, and oxidative stress further promotes tau pathology, leading to synaptic dysfunction and neuronal loss (2, 3). In addition, neuroinflammation plays a critical role in AD progression. Activated microglia and astrocytes release pro-inflammatory cytokines, including interleukin-1*α* (IL-1α), interleukin-1β (IL-1β), and tumor necrosis factor (TNF-α), which exacerbate neuronal injury and disease progression (3, 4). The gut–brain axis, a bidirectional communication network between the gut and the central nervous system (CNS), has attracted increasing attention in neurodegenerative diseases. Gut microbiota dysbiosis can increase intestinal permeability and promote systemic inflammation, facilitating the translocation of microbial metabolites and immune mediators into circulation (4). Emerging evidence indicates that dysbiosis of the gut microbiota in AD mice, which elevates phenylalanine and isoleucine levels, promotes Th1 cell differentiation and brain infiltration, triggering microglial activation and neuroinflammation (4). Therefore, gut microbiota dysbiosis is considered an important contributor to AD progression. Together, these factors interact to drive the development and progression of AD. Currently, AD treatment strategies primarily involve pharmacological interventions and acupuncture, which can only modestly delay disease progression but neither cure nor reverse the condition (4, 5). A growing body of research suggests that dietary factors may play an important role in reducing the risk of and slowing the progression of AD (6). Therefore, researchers are increasingly focusing on preventing or slowing AD through dietary interventions.

Compounds with biological activity from edible and medicinal fungi are thought to contribute significantly to preserving cognitive performance and protecting neuronal integrity (7–9). Ergothioneine (EGT) is a naturally occurring dietary antioxidant often called the “longevity vitamin” (10). Although humans cannot synthesize EGT endogenously, it is efficiently absorbed from the diet via the organic cation transporter novel type 1 (OCTN1), which is abundantly expressed in the gut and brain, thereby facilitating its accumulation in neural tissues (11). Studies have shown that dietary supplementation with EGT-rich *Pleurotus eryngi* extract improves cognitive function in Aβ-treated mice and reduces lipid peroxidation and protein carbonylation (12). These findings suggest that EGT-rich mushroom supplementation may help maintain neuronal function and mitigate neurodegeneration. *Hericium erinaceus* (*H. erinaceus*), a major edible and medicinal mushroom rich in EGT, has been extensively studied for its therapeutic potential in gastrointestinal disorders, diabetes, hyperlipidemia, neurodegenerative diseases, and cancer (13). Previous research has shown that *H. erinaceus* can enhance synaptic transmission at mossy fiber–CA3 connections in the hippocampus and improve motor performance, recognition memory, and neurogenesis in mice (10, 14). However, the EGT content in *H. erinaceus* mycelium is relatively low (0.0038–0.0469 mg/g DW) (15), potentially limiting its functional efficacy when consumed as food. Therefore, the effects of the EGT-enriched water extract of *H. erinaceus* as a dietary intervention for AD remain unclear.

In this study, a high EGT-producing *H. erinaceus* strain HE-17 was selected from 29 isolates. Its optimal culture conditions and amino acid supplementation were established through single-factor and orthogonal experiments, and the major bioactive components were quantified. In addition, its effects on the progression of AD pathology were evaluated in APP/PS1 mice. This study aimed to elucidate the potential of the water extract of *H. erinaceus* as a natural functional food ingredient for promoting brain health. Therefore, we hypothesized that the EGT-enriched water extract of *H. erinaceus* (WEH) could ameliorate cognitive impairment in AD by regulating oxidative stress, neuroinflammation, and gut microbiota composition.

## 2 Materials and methods

### 2.1 Strains and culture

A total of 29 strains of *H. erinaceus* (HE-1 to HE-29) were maintained in the Laboratory of Precision Nutrition and Health, College of Food Science, South China Agricultural University.

*H. erinaceus* strains were activated on potato dextrose agar (PDA) medium (potato 200 g/L, glucose 20 g/L, agar 20 g/L) and incubated at 25 °C under light until the mycelium fully covered the plates. The activated strains were then cultured in a screening medium (potato 200 g/L, glycerol 98 g/L, soybean flour 60 g/L, sucrose 20 g/L, MgSO_4_ 1.5 g/L, KH_2_PO_4_ 3 g/L, and FeSO_4_ 0.5 g/L) and incubated at 25 °C in the dark for 30 days. Strain screening was performed based on biomass and EGT content. The selected strains were subsequently re-inoculated onto PDA plates and cultured until the mycelium fully covered the surface. Agar plugs (approximately 0.5 cm × 0.5 cm) were then cut from the plates and used for subsequent inoculation. The agar plugs were inoculated into a wheat-based medium (wheat grain 100 g, glucose 1 g, water 100 mL, CaCO_3_ 1 g, peptone 1 g, and gypsum 1 g) and incubated at 29 °C in the dark until the mycelium fully colonized the substrate, yielding the wheat spawn. The wheat spawn was inoculated at 12% (w/w) into a basal fermentation medium containing 58% wheat bran and 42% soybean flour, followed by incubation at 30 °C to optimize the fermentation conditions of *H. erinaceus*.

### 2.2 Strain screening

Mycelia cultivated in the screening medium were harvested, oven-dried, and weighed, and then ground under liquid nitrogen for subsequent analysis. EGT content was rapidly determined using an enzymatic method as described by Zhou et al. (16). Strain screening was based on EGT production and biomass, and the optimal strain was used for subsequent experiments. EGT levels in the four selected strains were determined using high-performance liquid chromatography (HPLC) according to Huang et al. (17). Briefly, separation was performed on an Ultimate® HILIC Amphion II column (4.6 mm × 150 mm, 5 μm) using a mobile phase consisting of 80% acetonitrile and 20% ultrapure water. The detection wavelength was set at 254 nm, and the flow rate was 0.9 mL/min.

### 2.3 Optimization of the fermentation medium

Single-factor experiments were conducted to evaluate the effects of initial moisture content, fermentation time, and substrate loading on EGT production (mg/g DW) in *H. erinaceus* mycelium. Different water contents (30–100%, w/w), fermentation times (1–15 days after full mycelial colonization), and substrate loadings (10–70 g) were set, with fermentation conducted in 480 mL glass bottles at 30 °C using a 12% inoculum rate. Based on the single-factor results, a three-factor, four-level orthogonal design was subsequently carried out with water content, fermentation time, and substrate loading as factors.

Based on the optimized basal medium, single-factor experiments were conducted by supplementing different concentrations of histidine (0–12.5 mg/g), cysteine (0–4.5 mg/g), and methionine (0–12.5 mg/g) to evaluate their effects on EGT production. Subsequently, an orthogonal design (three-factor, five-level) was performed to investigate the combined effects of the three amino acids on EGT production.

After cultivation in the optimized medium, the fermented material (mycelia and substrate mixture) was harvested and dried at 60 °C to constant weight, and then ground into a powder. The powder was stored in a desiccator until further use.

### 2.4 Preparation and compositional analysis of WEH

The *H. erinaceus* HE-17 powder was extracted with water at a solid–liquid ratio of 1:20 (w/v), heated at 90 °C for 60 min, and then centrifuged at 8,000 × g for 20 min to collect the supernatant. The procedure was performed in duplicate, after which the collected supernatants were pooled, concentrated to 20% of the original volume using a rotary evaporator, and subsequently lyophilized to obtain WEH.

The concentrations of EGT, free amino acids, polysaccharides, triterpenes, phenols, and proteins in WEH were quantified according to the methods described by Huang et al. (17).

### 2.5 Animals and experimental design

Fifty-six male C57BL/6J mice (3 months old; body weight 24 ± 1 g) were purchased from Cyagen Biomodels (Guangzhou) Co., Ltd. (License No. SCXK (Yue) 2022-0016), including 40 APP/PS1 transgenic mice and 16 wild-type controls. The APP/PS1 model co-expresses a chimeric mouse/human amyloid precursor protein (Mo/HuAPP695swe) and a human PS1-dE9 mutation, both driven by the mouse prion protein promoter to ensure neuron-specific expression in the CNS. All mice were maintained at the Laboratory Animal Center of South China Agricultural University (License No. SYXK (Yue) 2024-0136) under controlled environmental conditions (25 ± 1 °C, 55–65% relative humidity) with a 12 h light/dark cycle (lights on from 07:00 to 19:00). All experimental protocols were reviewed and approved by the Animal Ethics Committee of South China Agricultural University (Approval No. 2024b105).

After a one-week acclimatization period, mice were allocated to their respective experimental groups using a computer-generated randomization sequence (Microsoft Excel). The APP/PS1 transgenic mice were randomly allocated into 4 groups (*n* = 10): model control group (AC); low-dose WEH-treated group (0.50 g/kg BW/d, with an EGT content of 1.44 mg/kg) (AL); high-dose WEH-treated group (2 g/kg BW/d, with an EGT content of 5.76 mg/kg) (AH); EGT-treated group (1.44 mg/kg BW/d) (ALE). Negative wild-type mice were divided into 2 groups (*n* = 8): wild-type control (NC) and high-dose WEH-treated group (2 g/kg BW/d, with an EGT content of 5.76 mg/kg) (NH). AC and NC were provided with equal doses of sterile water. The administered doses for the mouse experiments were set at 12 and 48 times (7.2 mg/d and 28.8 mg/d) the adult intake dose (assuming an adult body weight of 60 kg and a conversion factor of 12.33), according to the calculated method of Huang et al. (17). Each oral gavage was administered at a volume of 10 mL/kg of body weight. The administered solutions were prepared based on the administered dose and gavage volume, using sterile distilled water as the solvent on a clean bench. All treatments were administered once daily for 90 consecutive days.

During the test period, the mice had ad libitum access to food and water. Body weight was recorded every 10 days, and food intake was monitored daily. Behavioral observations were performed regularly. Fecal samples were collected 1 week before behavioral testing, and behavioral testing was conducted 10 days before tissue collection. Before euthanasia, mice were fasted for 12 h. Under anesthesia, blood was collected, and the eyes were subsequently enucleated, followed by euthanasia via cervical dislocation. Blood samples were allowed to stand at room temperature for 3 h, followed by centrifugation at 3,500 × g for 15 min at 4 °C to separate the serum, which was then stored at −80 °C. Brains were harvested and placed on dry ice, and three mice per group were randomly selected for analysis. One hemisphere was fixed in 4% paraformaldehyde for histology, and the hippocampus was dissected from the remaining tissue. Both hippocampal and remaining brain tissues were frozen in liquid nitrogen and stored at −80 °C for further analysis.

### 2.6 Behavioral experiment

Spatial learning and memory in APP/PS1 mice were assessed using the Morris Water Maze (MWM). The circular pool (120 cm in diameter, 50 cm in height) was filled with water to a depth of 30 cm at 22 ± 1 °C, and a hidden platform (10 cm in diameter) was submerged 0.5–1 cm below the water surface. Titanium dioxide was added to make the water opaque. The pool was divided into four quadrants (N, S, W, E) with eight entry points (Supplementary Figure 1). On day 1 (visible platform), the platform protruded 0.5–1 cm above water. Each mouse underwent four trials, with the platform placed in two different locations. Mice had 60 s to find the platform; if unsuccessful, they were guided to it and allowed to remain for 15 s. On days 2–6 (hidden platform), the platform was submerged 0.5–1 cm below the water surface in the E quadrant. Each mouse completed four trials per day, starting from four randomly selected entry points. On day 7 (probe test), the platform was removed, and mice were released from a fixed entry point (opposite the previous platform location), the farthest from it. Movements were tracked to evaluate memory retention.

### 2.7 Assessment of organ index, antioxidant activity and inflammatory factor

The liver, kidney, and spleen of each mouse were harvested and weighed to calculate the organ index. Tissue sections were collected for histological analysis: a cross-section from the tip of the liver lobe, an approximately pea-sized section from the left kidney, and a transverse section of the colon. These samples were processed and stained with hematoxylin and eosin (H&E) for pathological examination. Biochemical assays were performed using commercial kits to measure the levels of malondialdehyde (MDA), total antioxidant capacity (T-AOC), superoxide dismutase (SOD), catalase (CAT), nitric oxide (NO), glutathione (GSH), and acetylcholine (Ach) in the brain, liver, colon, and kidney tissues. In addition, ELISA kits were utilized to measure the concentrations of IL-1β, interleukin-6 (IL-6), and TNF-*α* in both serum and brain tissue samples. These kits were sourced from the Nanjing Jiancheng Bioengineering Institute.

### 2.8 Histology analysis

H&E staining and Nissl staining were conducted according to the method described by He et al. (18) to evaluate morphological and histopathological alterations in mouse brain tissue. Brain samples fixed in 4% paraformaldehyde were embedded in paraffin, sectioned at a thickness of 4 μm, and subsequently stained with hematoxylin/eosin and cresyl violet, respectively. Histopathological changes in brain tissue were observed using a light microscope. For each mouse, three coronal sections at comparable anatomical levels were selected for analysis. Representative images were collected, and regions of interest (ROIs) were defined in the hippocampus (cornu ammonis 1 [CA1], cornu ammonis 3 [CA3], and dentate gyrus [DG]) and cortex. For each section, three to five non-overlapping fields were randomly selected and imaged under identical conditions. All analyses were performed in a blinded manner.

### 2.9 Immunofluorescence

Following dewaxing and antigen retrieval, brain tissue sections were blocked with 10% donkey serum for 30 min at 37 °C. The sections were then incubated overnight at 4 °C with the following primary antibodies: Aβ (1:200, ab201060, Abcam, UK), Tau (1:200, ab206060, Abcam, UK), Iba1 (1:200, ab178846, Abcam, UK), and GFAP (1:200, ab68428, Abcam, UK). After thorough washing, the sections were incubated with a fluorophore-conjugated secondary antibody (1:400, A21206, Life Technologies, USA) for 45 min at 37 °C. The samples were then rinsed with Tris-buffered saline containing 0.1% Tween-20 (TBST), and the nuclei were counterstained with DAPI (1:500) for 5 min in the dark, followed by a final rinse with TBST. The sections were mounted with an anti-fade mounting medium and stored at 4 °C in the dark. Brain tissues were sectioned at 4 μm, and three coronal sections per mouse at comparable anatomical levels were analyzed. Immunofluorescence images were acquired under a fluorescence microscope using identical exposure settings. For each section, three to five non-overlapping fields were randomly selected for analysis. Fluorescence signals for Aβ, Tau, Iba1, and GFAP were quantified as mean fluorescence intensity using ImageJ software (National Institutes of Health, Bethesda, MD, USA), with consistent threshold settings applied across all groups. All image acquisition and analyses were performed in a blinded manner.

### 2.10 16S rRNA sequencing of gut microbiota

The DNA extraction kit (D6356-02, Magen, China) was used to extract fecal bacterial DNA. Genomic DNA was used as a template for PCR amplification with full-length 16S rRNA gene primers containing sample-specific barcodes. The amplified products were purified, quantified, and normalized to construct SMRTbell libraries, which were subjected to quality control prior to sequencing on the PacBio Sequel II platform (Pacific Biosciences, Menlo Park, CA, USA). Raw sequencing data were processed with SMRT Link to generate circular consensus sequencing (CCS) reads. CCS sequences were demultiplexed using Lima (v1.7.0), followed by primer trimming and length filtering using Cutadapt (v1.9.1). Chimeric sequences were identified and removed using UCHIME (v4.2) to obtain high-quality, effective CCS sequences. Feature classification based on amplicon sequence variants (ASVs), taxonomic annotation, and relative abundance profiling was subsequently performed. Alpha diversity indices, including Abundance-based Coverage Estimator (ACE), Chao1, Shannon, and Simpson, were calculated to evaluate microbial richness and diversity within samples. Beta diversity was evaluated using principal coordinate analysis (PCoA) based on Bray Curtis distance matrices to assess differences in microbial community structure among groups. The statistical significance of group separation was further tested using analysis of similarities (ANOSIM). These analyses were performed using QIIME 2 (version 2020.11).

### 2.11 Statistical analysis

All data are expressed as mean ± standard deviation (SD). Statistical analyses were performed using GraphPad Prism 10. Normality of data distribution was assessed using the Shapiro–Wilk test, and homogeneity of variance was evaluated using Levene’s test. Comparisons between two groups were performed using an independent samples *t*-test. For multiple-group comparisons, one-way analysis of variance (ANOVA) was conducted, followed by Tukey’s *post hoc* test. Statistical significance was defined as *p* < 0.05. Microbiota-related figures were generated using the Oebiotech cloud platform.^1^

## 3 Results

### 3.1 Screening of high-EGT *H. erinaceus* strains and medium optimization

**Figure 1:**
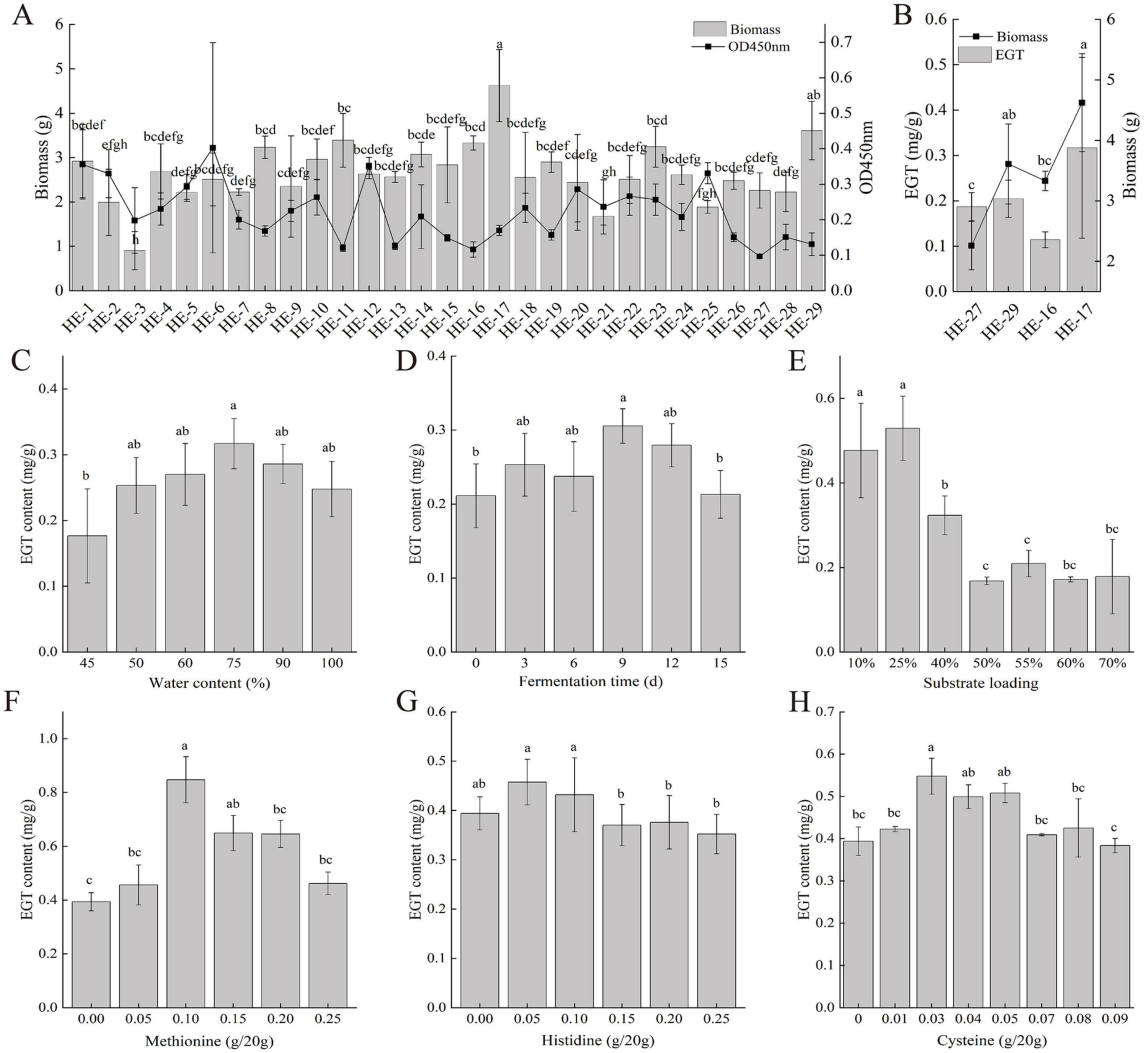
Screening of high-yield EGT strains **(A, B)** and optimization of cultivation conditions **(C–H)**. Different lowercase letters in panels **(A)** and **(B)** indicate significant differences in biomass among groups (*p* < 0.05), while different lowercase letters in panels **(C)–(H)** indicate significant differences among groups (*p* < 0.05).

Mycelial biomass and EGT content were used as the two screening criteria, and the results are shown in Figure 1A. A lower absorbance at 450 nm indicated a higher EGT content, while a higher biomass value reflected better growth performance. Based on the combined evaluation of these two parameters, four strains were selected for subsequent experiments, ranked as *H. erinaceus* HE-17, *H. erinaceus* HE-29, *H. erinaceus* HE-27, and *H. erinaceus* HE-16. Furthermore, the EGT content in the mycelia of these four strains was quantified by HPLC, as shown in Figure 1B. The ranking was largely consistent with that obtained from the initial screening. Therefore, *H. erinaceus* HE-17 was selected for subsequent experiments.

To further enhance EGT production during the fermentation of *H. erinaceus* HE-17, single-factor experiments were conducted to optimize medium water content, fermentation time, substrate loading, and the concentrations of precursor amino acids for EGT biosynthesis (Met, His, Cys). As shown in Figures 1C–E, the highest EGT content was achieved at a medium water content of 75%, on day 9 after the mycelia fully colonized the substrate, and at a loading amount of 25%. Based on these results, an orthogonal experiment with three factors and four levels was designed, with the factor levels presented in Supplementary Table 1. The results indicated that the optimal combination for *H. erinaceus* HE-17 was C1B1A1 (Supplementary Table 2), corresponding to a loading amount of 10 g, a moisture content of 60%, and a fermentation time of 6 days. Met, His, and Cys are three key substrates in the EGT biosynthetic pathway. Therefore, under the optimized conditions described above, the concentrations of these amino acids were further optimized using single-factor experiments. As shown in Figures 1F–H, the highest EGT content was achieved at supplementation levels of 0.1 g/20 g for Met, 0.05 g/20 g for His, and 0.03 g/20 g for Cys. Based on these results, a three-factor, four-level orthogonal design was further conducted, with the factor levels presented in Supplementary Table 3. The orthogonal analysis (Supplementary Table 4) indicated that the optimal combination was A4C1B3, corresponding to 0.20 g/20 g Met, 0.01 g/20 g Cys, and 0.10 g/20 g His. Accordingly, the final optimized conditions for high EGT production by *H. erinaceus* HE-17 were as follows: a loading amount of 10 g, a moisture content of 60%, and an extended fermentation time of 6 days. The optimal amino acid combination was 0.20 g/20 g Met, 0.01 g/20 g Cys, and 0.10 g/20 g His.

The content of various components in the *H. erinaceus* HE-17 fungal substance and WEH is shown in Table 1. The EGT content in WEH reached 2.57 ± 0.14 mg/g DW, which was approximately 2.4-fold higher than that of the fungal substance (1.07 ± 0.06 mg/g DW) and was substantially higher than that reported for primordium extracts (1.3 ± 0.57 mg/g DW) (19). Notably, WEH exhibited higher levels of total phenolics and free amino acids compared to the fungal substance, indicating that the extraction process enhanced the release and solubility of these bioactive components.

**Table 1:** Composition content of *H. erinaceus* HE-17 and WEH (mean ± SD, *n* = 3).

Component	Content in WEH (mg/g)	Content in *H. erinaceus* HE-17 (mg/g)
EGT	2.57 ± 0.14	1.07 ± 0.06
Triterpenoid	58.67 ± 0.53	24.52 ± 6.52
Phenol	2.07 ± 0.13	8.63 ± 0.05
Crude polysaccharide	6.50 ± 21.72	27.21 ± 4.34
Protein	12.64 ± 1.10	10.57 ± 1.48
Free amino acid	70.08 ± 3.94	29.29 ± 3.08

WEH, water extract of *Hericium erinaceus* HE-17; EGT, ergothioneine.

### 3.2 Effect of WEH on body weight and organs in mice

**Figure 2:**
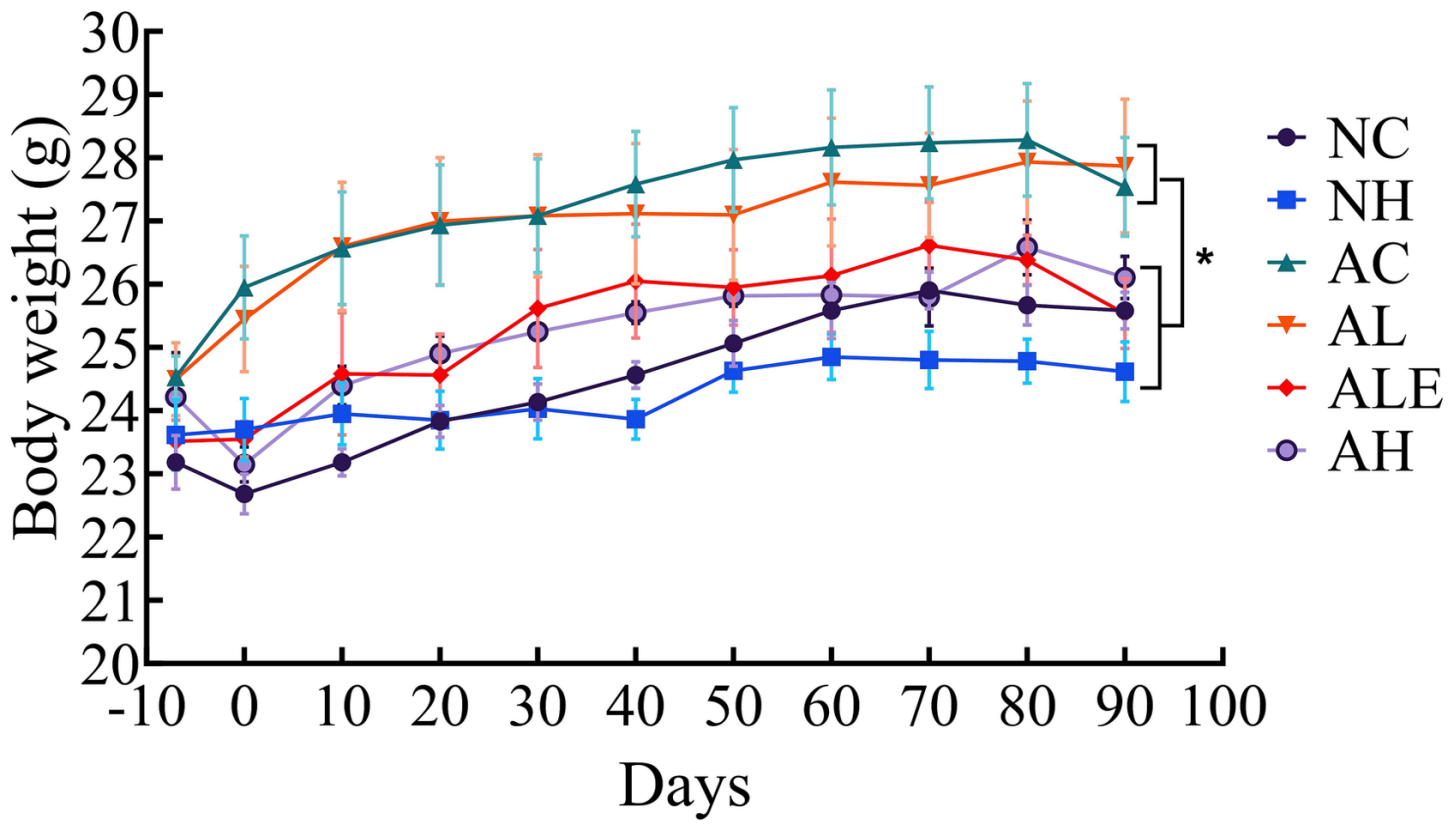
Effect of WEH on body weight in C57BL/6J mice (mean ± SD, *n* = 8–10). * indicates a significant difference between the indicated groups (*p* < 0.05). WEH, water extract of *Hericium erinaceus* HE-17; NC, wild-type control group; NH, wild-type mice treated with high-dose WEH (2 g/kg BW/d); AC, APP/PS1 model control group; AL, APP/PS1 mice treated with low-dose WEH (0.50 g/kg BW/d); AH, APP/PS1 mice treated with high-dose WEH (2 g/kg BW/d); ALE, APP/PS1 mice treated with ergothioneine (1.44 mg/kg BW/d).

Body weight was monitored throughout the experiment to assess the effects of WEH on mice (Figure 2). All groups showed progressive weight gain, with a slight decline after day 80, likely due to behavioral testing. APP/PS1 mice had significantly higher body weights than wild-type mice (*p* < 0.05). Furthermore, the AH and ALE groups had body weight trends similar to those of wild-type controls, while the AC and AL groups maintained higher body weights. High-dose WEH supplementation in NH did not affect body weight (*p* > 0.05), indicating no adverse effects on normal growth.

The organ index serves as an indicator of potential organ damage in mice. As shown in Table 2, the liver, kidney, and spleen indices did not differ significantly between APP/PS1 mice and wild-type controls, indicating that AD progression had no evident impact on the growth and development of these organs. Additionally, high-dose WEH did not exhibit noticeable toxicity or inhibitory effects on the normal growth and development of the liver, spleen, and kidney.

**Table 2:** Effect of WEH on organ indices in mice (mean ± SD, *n* = 8–10).

Groups	Liver index (%)	Kidney index (%)	Spleen index (%)
NC	3.62 ± 0.30	1.15 ± 0.09	0.21 ± 0.05
NH	3.63 ± 0.29	1.20 ± 0.19	0.23 ± 0.07
AC	3.76 ± 0.18	1.04 ± 0.06	0.23 ± 0.05
AL	3.49 ± 0.43	1.12 ± 0.09	0.22 ± 0.03
ALE	3.54 ± 0.15	1.09 ± 0.06	0.18 ± 0.04
AH	3.55 ± 0.14	1.10 ± 0.08	0.19 ± 0.03

WEH, water extract of *Hericium erinaceus* HE-17; NC, wild-type control group; NH, wild-type mice treated with high-dose WEH (2 g/kg BW/d); AC, APP/PS1 model control group; AL, APP/PS1 mice treated with low-dose WEH (0.50 g/kg BW/d); AH, APP/PS1 mice treated with high-dose WEH (2 g/kg BW/d); ALE, APP/PS1 mice treated with ergothioneine (1.44 mg/kg BW/d).

To further evaluate the effects of WEH on organ integrity, histopathological examination was performed using H&E-stained tissue sections. As shown in Supplementary Figure 2, liver cells in the NH group were well-preserved and orderly arranged. The renal cortex exhibited normal glomerular morphology and size, while the renal medulla showed uniformly distributed tubular epithelial cells with intact structure and no apparent damage. The colonic epithelium also displayed a normal number of goblet cells, comparable to that observed in the NC group. Moreover, no significant histopathological differences were observed in the liver, kidney, and colon between APP/PS1 mice and wild-type controls. These findings suggest that WEH supplementation does not induce noticeable toxic effects on the structural integrity of these organs in either model or normal mice.

### 3.3 Effects of WEH supplementation on the locomotor and exploratory activities in APP/PS1 mice

**Figure 3:**
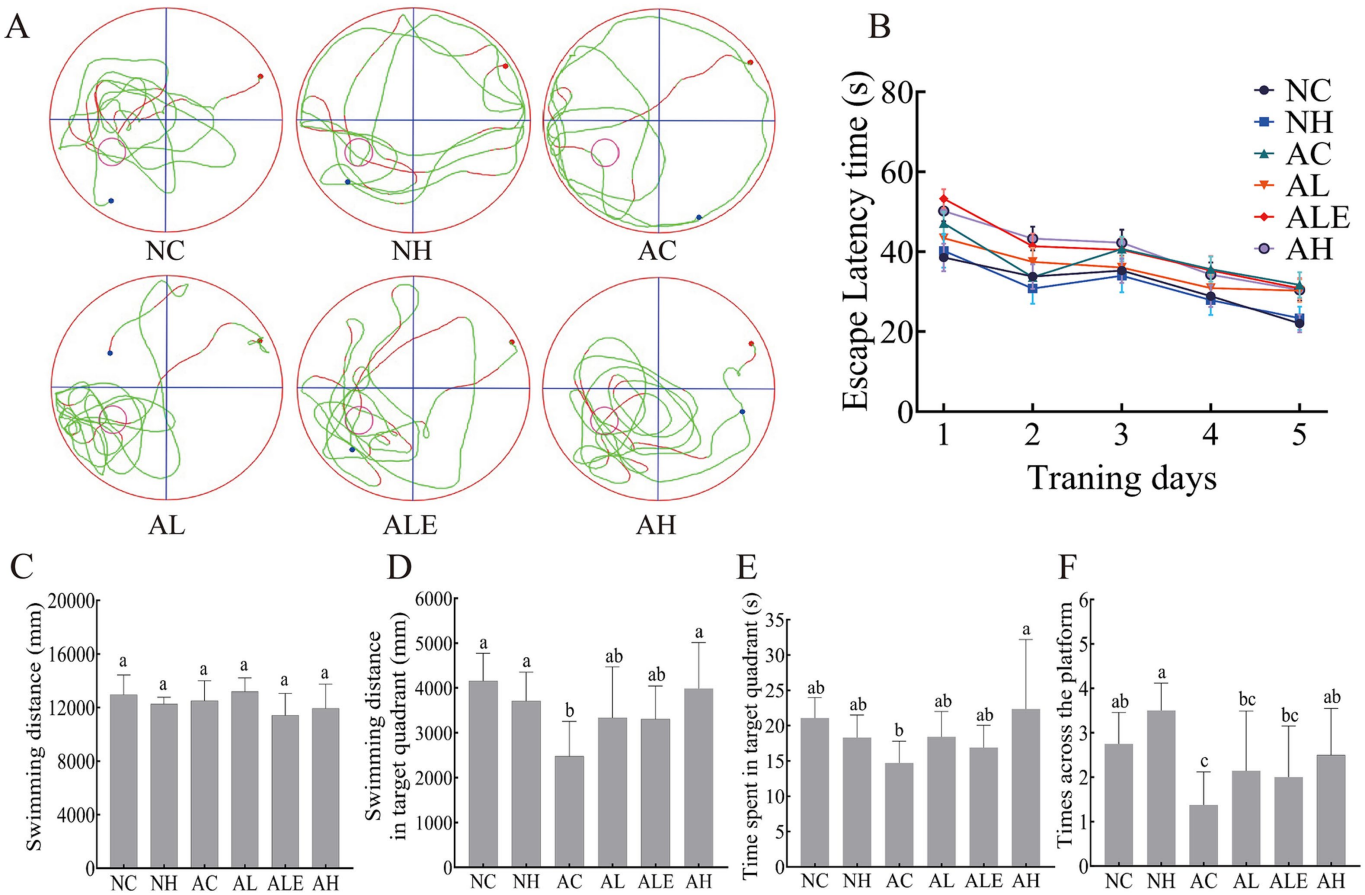
Morris water maze (MWM) tests in C57BL/6J mice (mean ± SD, *n* = 8–10). **(A)** Space exploration of representative locomotor trajectories in mice during the MWM test period. **(B)** Escape latency time in mice. **(C)** Total distance travelled by mice. **(D)** Distance mice moved in the quadrant where the station was located. **(E)** Time spent by mice in the quadrant where the station was located. **(F)** Number of times mice crossed the station. Different lowercase letters indicate significant differences between groups (*p* < 0.05). WEH, water extract of *Hericium erinaceus* HE-17; NC, wild-type control group; NH, wild-type mice treated with high-dose WEH (2 g/kg BW/d); AC, APP/PS1 model control group; AL, APP/PS1 mice treated with low-dose WEH (0.50 g/kg BW/d); AH, APP/PS1 mice treated with high-dose WEH (2 g/kg BW/d); ALE, APP/PS1 mice treated with ergothioneine (1.44 mg/kg BW/d).

To assess spatial learning and memory abilities in mice, the MWM was conducted. During the 5-day training period, the escape latency of mice across all groups decreased over time, indicating progressive learning, with no significant differences among groups (Figure 3B). The AC group tended to exhibit longer escape latencies than the other groups. Representative swimming trajectories are shown in Figure 3A. Wild-type mice in both the NC and NH groups predominantly navigated within the target quadrant containing the platform, exhibiting a higher frequency of platform crossings. In contrast, model mice in the AC group displayed a preference for swimming along the pool edges, with their movement patterns appearing more scattered. Following WEH treatment, mice in the AL and AH groups showed focused exploration concentrated in the target quadrant, with more streamlined trajectories. Although the ALE group exhibited somewhat more dispersed movement paths, there was still a notable increase in time spent in the target quadrant and in the number of platform crossings compared to the AC group.

Additionally, behavioral assessments were conducted after 90 consecutive days of WEH administration. The distance travelled by the mice in each group was basically the same during the testing period and did not differ significantly (*p* > 0.05) (Figure 3C). Compared with the wild-type NC group, APP/PS1 mice in the AC group showed marked impairments in spatial learning and memory. These deficits were reflected by a significant decrease in the number of platform crossings (from 2.75 ± 0.70 to 1.38 ± 0.74, *p* < 0.05) (Figure 3F), along with reduced time spent in the target quadrant (from 21.07 ± 2.89 s to 14.71 ± 3.09 s) (Figure 3E) and a shorter travel distance within that region (from 4,159.15 ± 611.64 mm to 2,476.56 ± 777.39 mm) (Figure 3D). In contrast, supplementation with WEH led to a progressive improvement in movement patterns in the model mice, characterized by more defined swimming trajectories and a preferential localization within the platform-containing quadrant. Compared with the AC group, animals in the AL group (18.39 ± 3.61 s; 3,336.95 ± 1,134.06 mm) and AH group (22.36 ± 9.83 s; 3,987.55 ± 1,027.25 mm) displayed substantial increases in both residence time and travel distance within the target quadrant, suggesting that WEH effectively ameliorated deficits in spatial learning and memory in APP/PS1 mice.

### 3.4 Effects of WEH on hippocampal neurons in APP/PS1 mice

**Figure 4:**
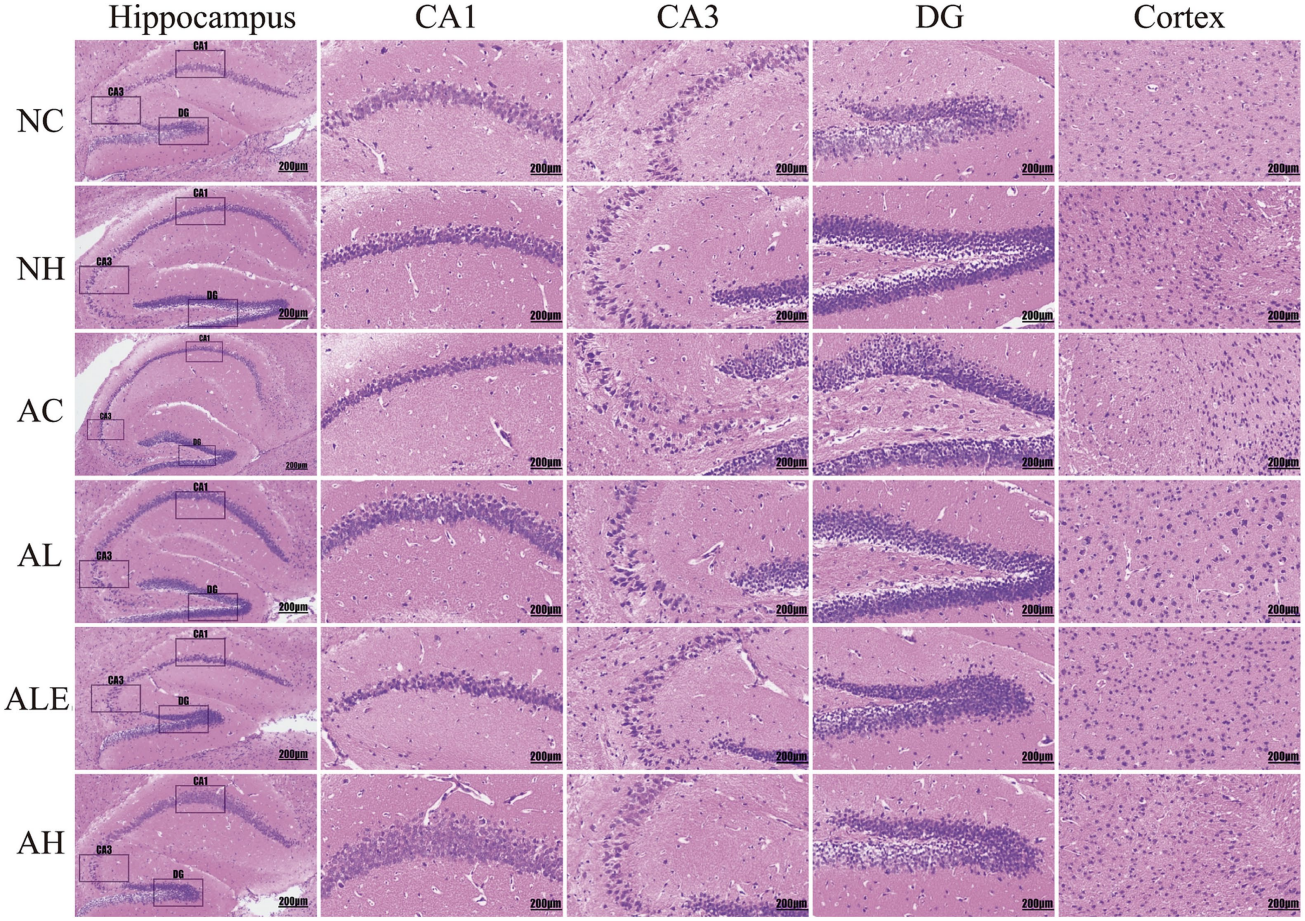
H&E staining of mouse hippocampus. CA1, CA3, and DG are the three subdivisions of the hippocampus. WEH, water extract of *Hericium erinaceus* HE-17; NC, wild-type control group; NH, wild-type mice treated with high-dose WEH (2 g/kg BW/d); AC, APP/PS1 model control group; AL, APP/PS1 mice treated with low-dose WEH (0.50 g/kg BW/d); AH, APP/PS1 mice treated with high-dose WEH (2 g/kg BW/d); ALE, APP/PS1 mice treated with ergothioneine (1.44 mg/kg BW/d).

**Figure 5:**
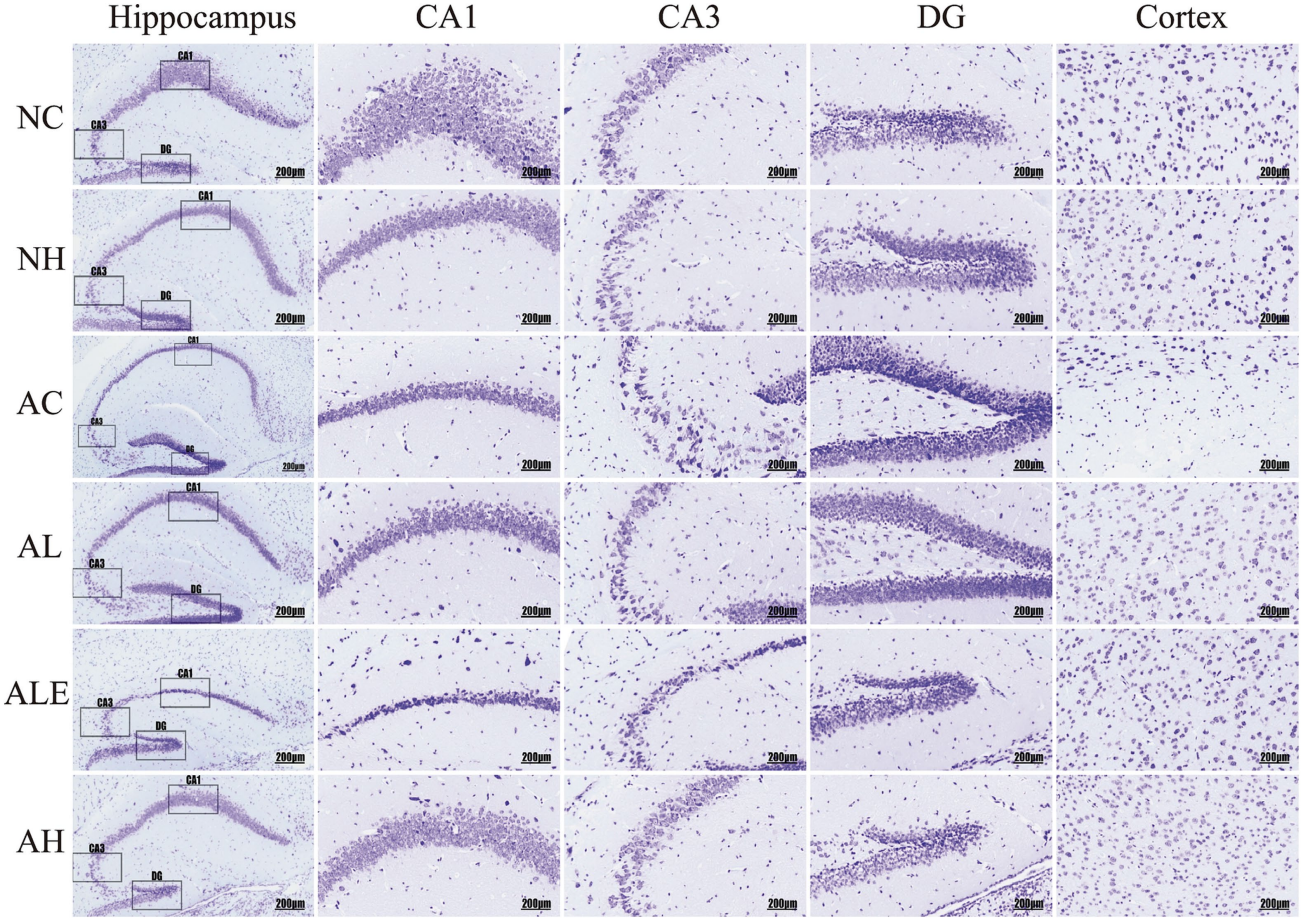
Nissl staining of mouse hippocampus. CA1, CA3, and DG are the three subdivisions of the hippocampus. WEH, water extract of *Hericium erinaceus* HE-17; NC, wild-type control group; NH, wild-type mice treated with high-dose WEH (2 g/kg BW/d); AC, APP/PS1 model control group; AL, APP/PS1 mice treated with low-dose WEH (0.50 g/kg BW/d); AH, APP/PS1 mice treated with high-dose WEH (2 g/kg BW/d); ALE, APP/PS1 mice treated with ergothioneine (1.44 mg/kg BW/d).

To evaluate WEH’s effects on the hippocampal and cortical regions of mice, H&E and Nissl staining were used to examine neuronal cell morphology and Nissl body distribution, as shown in Figures 4, 5. In wild-type mice, neurons in the CA1, CA3, and DG, as well as in the cortical regions of the NC group, were densely packed and well organized, exhibiting prominent nuclei and clearly defined boundaries (Figure 4). Model control mice in the AC group displayed distinct pathological changes, including nuclear condensation in the CA1 region, enlarged intercellular spaces, and disorganized neuronal arrangements in the CA3 and DG regions, as well as uneven cellular distribution in the cortical region, compared with the NC group. Following WEH supplementation, mice in the AL group exhibited notable restoration of neuronal structural integrity, with hippocampal and cortical neuronal morphology resembling that of the NC group. Further improvements were observed in the AH group, where high-dose WEH treatment led to better-preserved neuronal morphology, reduced neuronal damage, and a more orderly arrangement of hippocampal and cortical cells compared with the AC group. Nissl staining (Figure 5) revealed that, in comparison with the wild-type NC group, mice in the AC group displayed a marked decrease in Nissl bodies within the CA1 region and cortex. In the CA3 region, neuronal structures were disrupted and arranged irregularly, while neurons in the DG region displayed shrinkage, nuclear hyperchromasia, cytoplasmic dehydration, and vacuolar degeneration. After WEH supplementation, hippocampal neurons and cortical cell structures were improved in the AL, AH, and ALE groups, with the AH group showing the greatest improvement.

### 3.5 Effects of WEH treatment on Aβ deposition, tau tangles, IBA1, and GFAP in brain tissue of APP/PS1 mice

**Figure 6:**
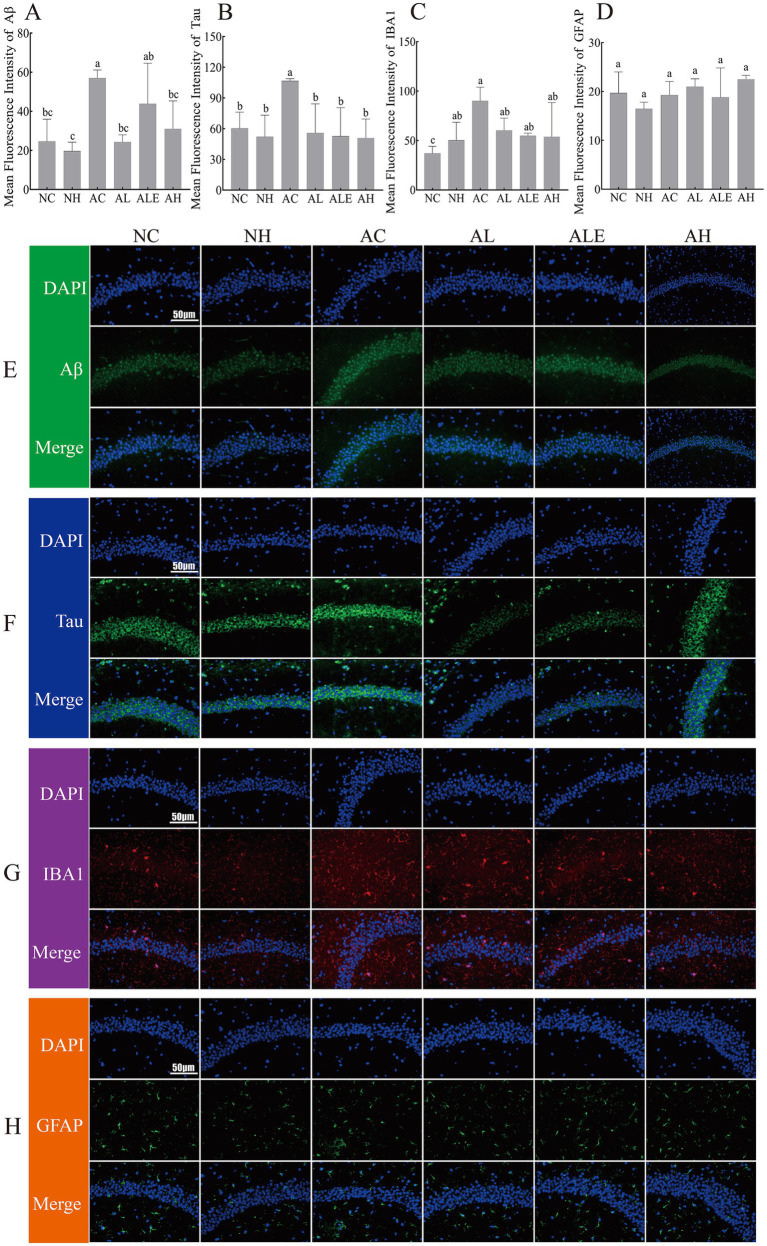
Effects of WEH treatment on Aβ deposition **(A, E)**, Tau junctions **(B, F)**, IBA1 **(C, G)**, and GFAP **(D, H)** in brain tissue of APP/PS1 mice (mean ± SD, *n* = 3). Different lowercase letters indicate significant differences between groups (*p* < 0.05). WEH, water extract of *Hericium erinaceus* HE-17; NC, wild-type control group; NH, wild-type mice treated with high-dose WEH (2 g/kg BW/d); AC, APP/PS1 model control group; AL, APP/PS1 mice treated with low-dose WEH (0.50 g/kg BW/d); AH, APP/PS1 mice treated with high-dose WEH (2 g/kg BW/d); ALE, APP/PS1 mice treated with ergothioneine (1.44 mg/kg BW/d).

Aβ accumulation in the brain and excessive tau phosphorylation are recognized as defining pathological features of AD. As shown in Figure 6A, hippocampal Aβ levels were significantly elevated in the AC group compared with the NC group, validating the successful induction of AD pathology in the model mice. Notably, treatment with WEH markedly reduced hippocampal Aβ expression in the AL, ALE, and AH groups (*p* < 0.05), suggesting its potential in mitigating amyloid pathology. Consistently (Figure 6B), the formation of neurofibrillary tangles was markedly elevated in the AC group relative to the NC group. Following WEH intervention, tau pathology was significantly alleviated in all treated groups (AL, ALE, and AH). To corroborate these findings, immunofluorescence staining was performed. The results revealed that WEH supplementation attenuated Aβ immunoreactivity (Figure 6E) and ameliorated tau pathology (Figure 6F) in the hippocampus of APP/PS1 mice.

Ionized calcium-binding adapter molecule 1 (IBA1) serves as a classical marker of microglial activation. As depicted in Figure 6C, hippocampal IBA1 expression was significantly increased in the AC group relative to the NC group, reflecting pronounced microglial activation in the model mice. Although IBA1 expression showed a slight downward trend following treatment, the difference was not statistically significant (*p* > 0.05). Glial fibrillary acidic protein (GFAP), a well-established marker of astrocyte activation, showed no significant difference in expression between wild-type and model mice, as presented in Figure 6D. Consistently, immunofluorescence analysis suggested a reduction in IBA1 immunoreactivity in the hippocampus following WEH treatment (Figure 6G), while GFAP expression remained largely unchanged (Figure 6H).

### 3.6 Effects of WEH on inflammation in APP/PS1 mice

**Figure 7:**
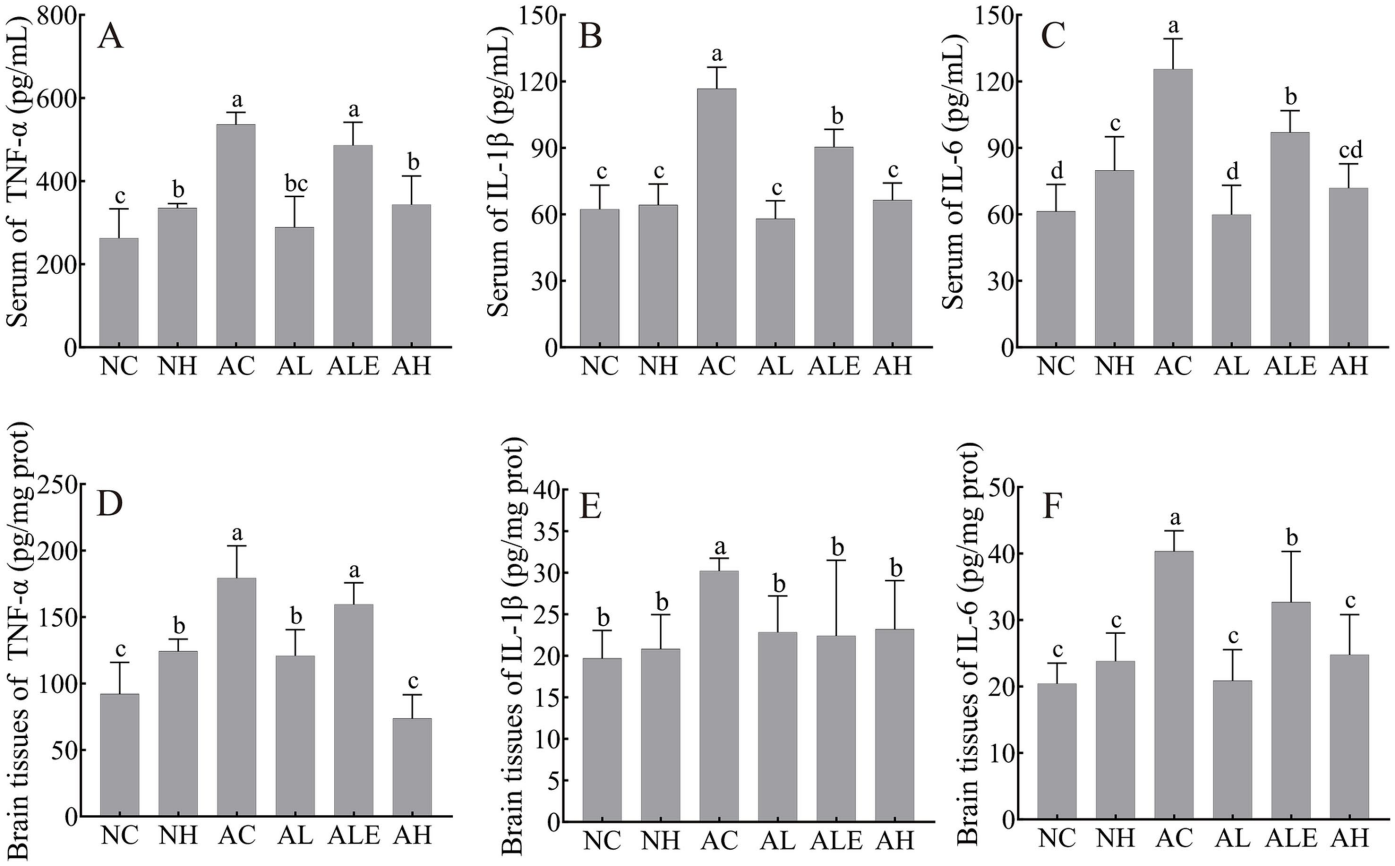
Content of inflammatory factors in brain and serum of APP/PS1 mice (mean ± SD, *n* = 8–10). **(A)** Content of TNF-*α* in serum. **(B)** Content of IL-6 in serum. **(C)** Content of IL-1β in serum. **(D)** Content of TNF-α in brain. **(E)** Content of IL-6 in brain. **(F)** Content of IL-1β in brain. Different lowercase letters indicate significant differences between groups (*p* < 0.05). WEH, water extract of *Hericium erinaceus* HE-17; NC, wild-type control group; NH, wild-type mice treated with high-dose WEH (2 g/kg BW/d); AC, APP/PS1 model control group; AL, APP/PS1 mice treated with low-dose WEH (0.50 g/kg BW/d); AH, APP/PS1 mice treated with high-dose WEH (2 g/kg BW/d); ALE, APP/PS1 mice treated with ergothioneine (1.44 mg/kg BW/d). TNF-α, tumor necrosis factor-α; IL-6, interleukin-6; IL-1β, interleukin-1β.

To assess the anti-inflammatory effects of WEH, the levels of key pro-inflammatory cytokines, including IL-1β, IL-6, and TNF-*α*, were measured in both serum and brain tissues of mice (Figure 7). In the serum, the concentrations of TNF-α (Figure 7A), IL-1β (Figure 7B), and IL-6 (Figure 7C) were significantly elevated in the AC group compared to the NC group (*p* < 0.05). Following WEH supplementation, pro-inflammatory cytokine levels were markedly reduced in both the AL and AH groups relative to the AC group. Although a modest reduction in TNF-α levels was observed in the ALE group compared with the AC group, the difference did not reach statistical significance. In contrast, administration of pure EGT resulted in a pronounced decrease in IL-1β and IL-6 levels (Figures 7B, C). Comparable anti-inflammatory trends were observed in brain tissue analyses (Figures 7D–F), with the AH group demonstrating a notably stronger reduction in TNF-α compared to the AL group (Figure 7D). These results demonstrate that both pure EGT and WEH effectively attenuate inflammatory responses in AD model mice, with WEH exhibiting superior anti-inflammatory efficacy.

### 3.7 Effects of WEH on oxidative stress in APP/PS1 mice

**Figure 8:**
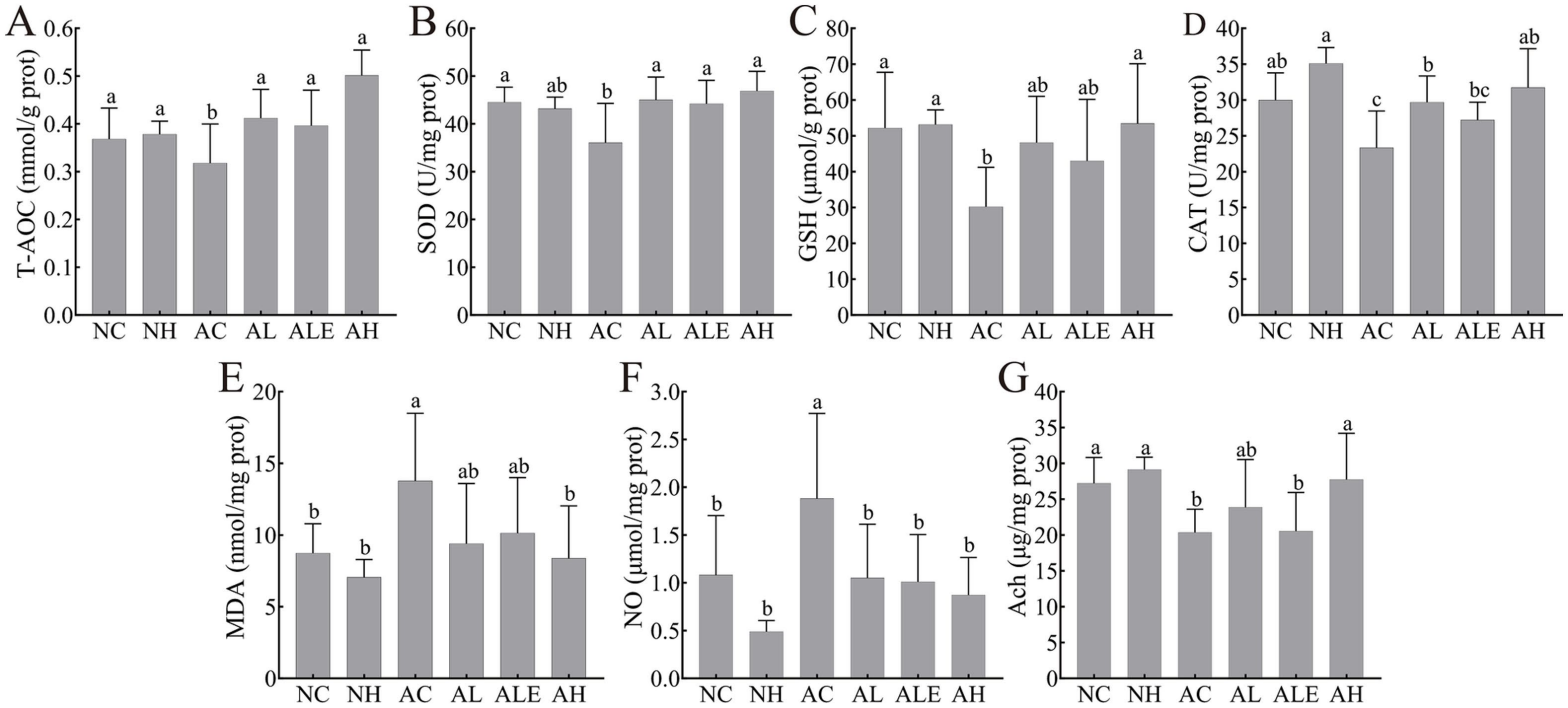
Effect of HSW on T-AOC **(A)**, SOD **(B)**, GSH **(C)**, CAT **(D)**, MDA **(E)**, NO **(F)**, and Ach **(G)** levels in APP/PS1 mice brain tissue (mean ± SD, *n* = 8–10). Different lowercase letters indicate significant differences between groups (*p* < 0.05). WEH, water extract of *Hericium erinaceus* HE-17; NC, wild-type control group; NH, wild-type mice treated with high-dose WEH (2 g/kg BW/d); AC, APP/PS1 model control group; AL, APP/PS1 mice treated with low-dose WEH (0.50 g/kg BW/d); AH, APP/PS1 mice treated with high-dose WEH (2 g/kg BW/d); ALE, APP/PS1 mice treated with ergothioneine (1.44 mg/kg BW/d). T-AOC, total antioxidant capacity; SOD, superoxide dismutase; GSH, glutathione; CAT, catalase; MDA, malondialdehyde; NO, nitric oxide; Ach, acetylcholine.

To investigate the neuroprotective potential of WEH in mitigating oxidative damage in the brain tissue of APP/PS1 transgenic mice, we measured several oxidative stress and antioxidant indicators, including T-AOC, SOD, GSH, CAT, MDA, NO, and Ach, and the results are shown in Figure 8. T-AOC (Figure 8A), SOD (Figure 8B), GSH (Figure 8C), and CAT (Figure 8D) were significantly lower in the AC group than in the NC group (*p* < 0.05), indicating a compromised antioxidant defense system. Supplementation with WEH significantly enhanced T-AOC, SOD, and CAT activities in model mice, while a marked increase in GSH was detected exclusively in the AH group. The concentrations of MDA (Figure 8E) and NO (Figure 8F) were markedly higher in the brain tissues of the AC group than in the NC group, reflecting heightened oxidative damage. WEH treatment reduced MDA levels across the AL, ALE, and AH groups, with the AH group showing the most pronounced reduction. Similarly, NO levels were significantly lower in all treatment groups than in the AC group, although no significant differences were observed among the AL, ALE, and AH groups (*p* > 0.05). For Ach (Figure 8G), the AC group exhibited a pronounced decline compared to the NC group. Following WEH supplementation, a significant elevation in Ach was observed only in the AH group, whereas no appreciable changes were observed in the AL and ALE groups.

### 3.8 Effects of WEH on the gut microbiota in APP/PS1 mice

To determine the effect of WEH on the gut microbiota of APP/PS1 mice, the fecal microbiota of mice in different treatment groups was analyzed. Analysis of alpha diversity metrics, including the ACE, Simpson, Chao1, and Shannon indices (Supplementary Table 5), revealed no statistically significant differences in bacterial richness or diversity between APP/PS1 model mice and the wild-type group (*p* > 0.05). Notably, WEH supplementation significantly enriched the diversity and abundance of microbiota in wild-type mice. According to ANOSIM analysis, within-group differences were smaller than between-group differences (Supplementary Table 6). Furthermore, PCoA showed a clear separation between the fecal microbiota profiles of model and wild-type mice, suggesting that APP/PS1 mice harbor distinct microbial communities compared to their wild-type counterparts (Supplementary Figure 3).

**Figure 9:**
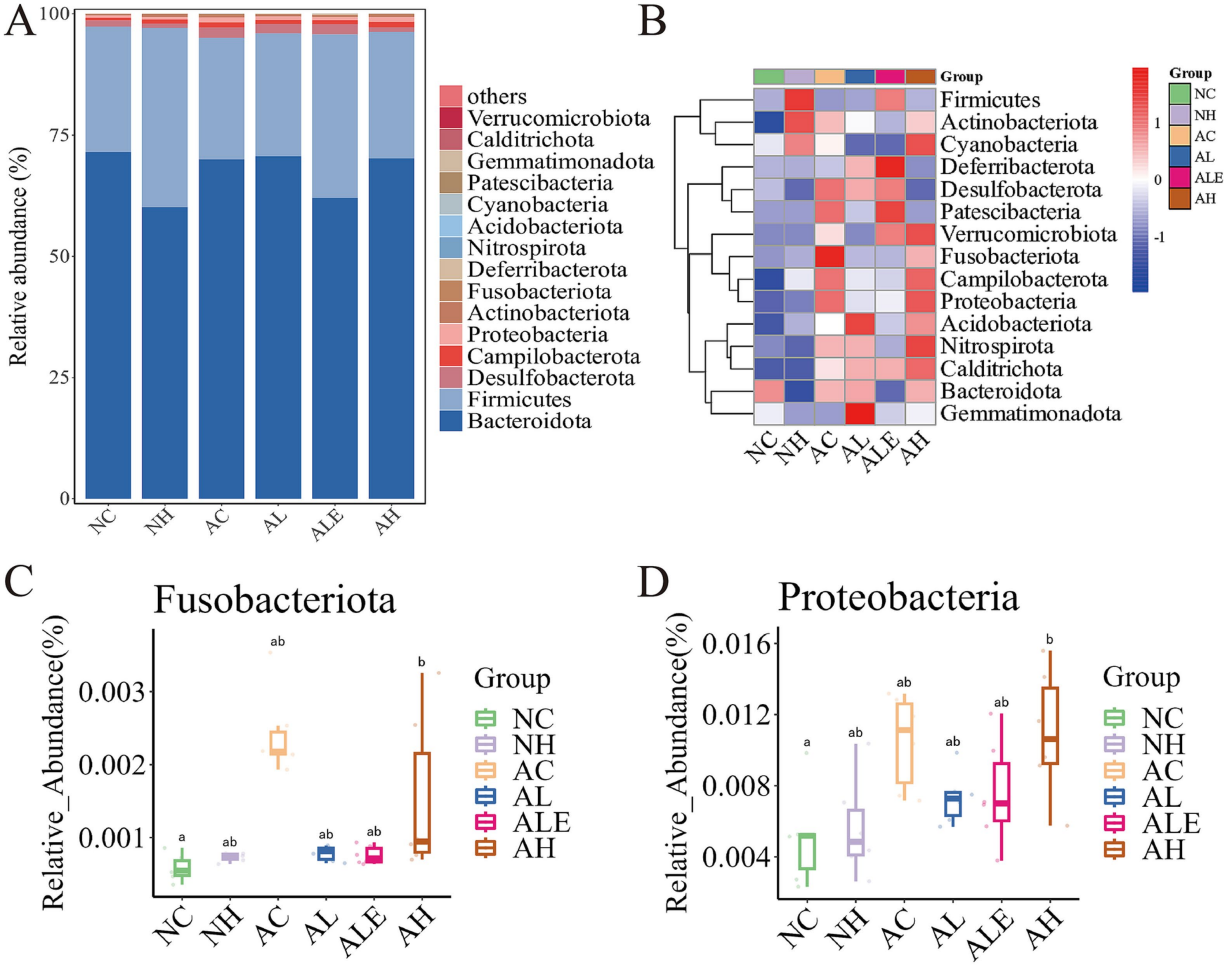
Relative abundance of gut microbiota at the phylum level in each group of mice. **(A)** Histogram of community structure. **(B)** Heatmap plot based on relative abundance. **(C, D)** Boxplot analysis of the relative abundance of two significantly different phyla. Different lowercase letters indicate significant differences between groups (*p* < 0.05). WEH, water extract of *Hericium erinaceus* HE-17; NC, wild-type control group; NH, wild-type mice treated with high-dose WEH (2 g/kg BW/d); AC, APP/PS1 model control group; AL, APP/PS1 mice treated with low-dose WEH (0.50 g/kg BW/d); AH, APP/PS1 mice treated with high-dose WEH (2 g/kg BW/d); ALE, APP/PS1 mice treated with ergothioneine (1.44 mg/kg BW/d).

**Figure 10:**
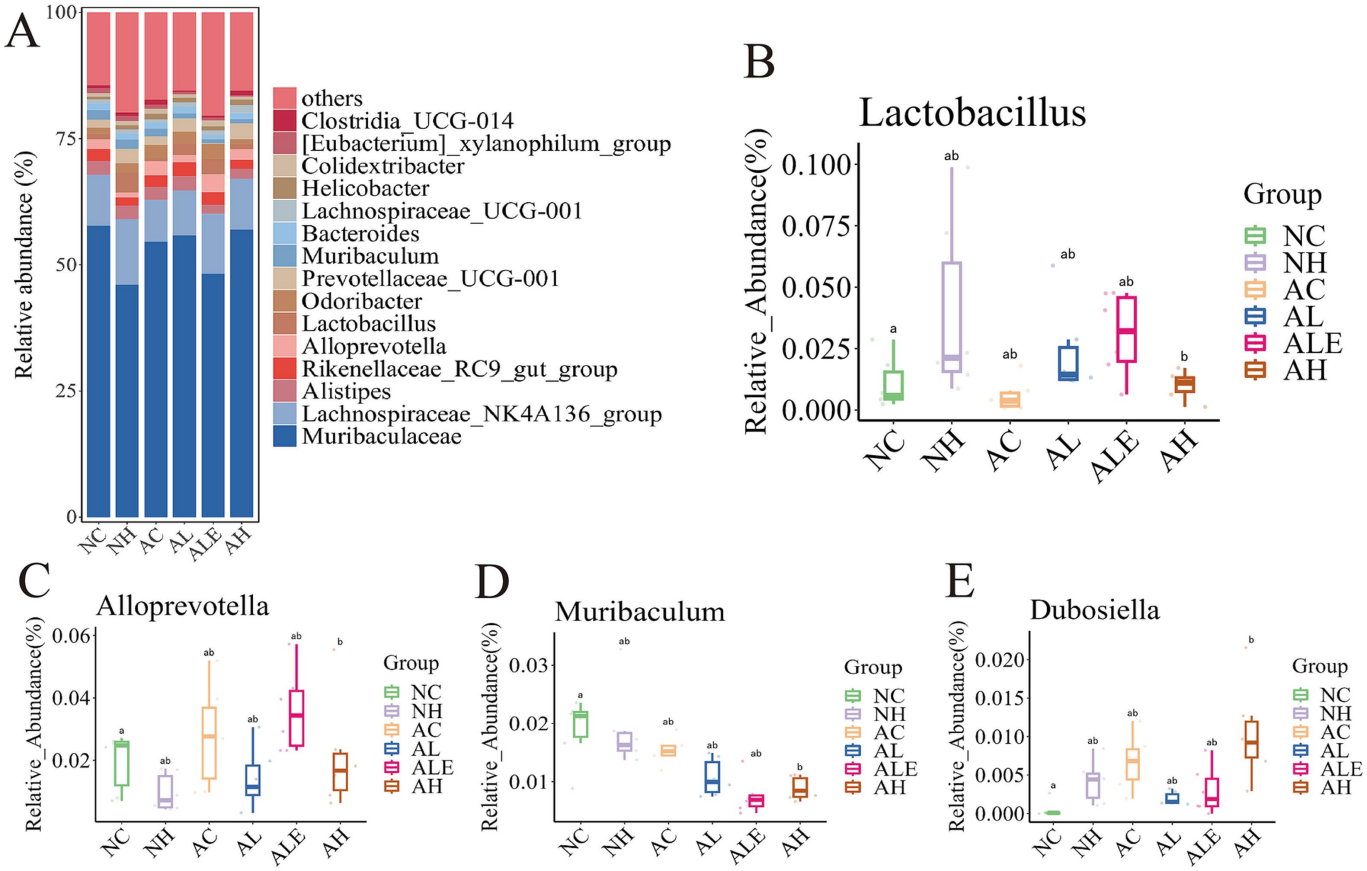
Relative abundance of gut microbiota at the genus level in each group of mice. **(A)** Histogram of community structure. **(B–E)** Boxplot analysis of the relative abundance of four significantly different genera. Different lowercase letters indicate significant differences between groups (*p* < 0.05). WEH, water extract of *Hericium erinaceus* HE-17; NC, wild-type control group; NH, wild-type mice treated with high-dose WEH (2 g/kg BW/d); AC, APP/PS1 model control group; AL, APP/PS1 mice treated with low-dose WEH (0.50 g/kg BW/d); AH, APP/PS1 mice treated with high-dose WEH (2 g/kg BW/d); ALE, APP/PS1 mice treated with ergothioneine (1.44 mg/kg BW/d).

At the phylum level, *Bacteroidota*, *Firmicutes*, *Desulfobacterota*, and *Campilobacterota* constituted the dominant gut microbiota in mice (Figure 9A). Furthermore, following WEH supplementation, the abundance of microbiota in the AL, ALE, and AH groups of model mice was obviously higher than that in the AC group (Figure 9B). Among these phyla, the most pronounced changes were observed in *Fusobacteriota* (Figure 9C) and *Proteobacteria* (Figure 9D). Specifically, the proportion of *Fusobacteriota* and *Proteobacteria* was markedly higher in the AC group of APP/PS1 model mice compared to the NC group of wild-type mice, indicating that AD pathology may be associated with the expansion of these potentially harmful phyla. Furthermore, compared with the AC group, the relative abundances of *Fusobacteriota* and *Proteobacteria* were lower in the AL and ALE groups. At the genus level, the composition and relative abundance of dominant gut microbiota varied notably among the different treatment groups (Figure 10A). The seven most abundant genera included *Muribaculaceae*, *Lachnospiraceae* NK4A136 group, *Alistipes*, *Rikenellaceae* RC9 gut group, *Alloprevotella*, *Lactobacillus*, and *Odoribacter*. Notably, *Alloprevotella*, *Muribaculum*, *Dubosiella* and *Lactobacillus* exhibited significant intergroup differences (Figures 10B–E). Specifically, pure EGT supplementation significantly increased *Alloprevotella*, and high-dose WEH enhanced *Dubosiella* abundance. Both WEH and EGT treatments reduced *Muribaculum*. Interestingly, low-dose WEH and pure EGT treatments promoted a notable increase in *Lactobacillus*.

## 4 Discussion

The WEH contained 2.57 ± 0.14 mg/g DW of EGT, substantially higher than that previously reported in primordium extracts (1.3 ± 0.57 mg/g DW) (19). In this study, high-dose WEH supplementation in NH did not affect body weight and was not associated with significant toxicity or adverse effects on the normal growth and development of the liver, spleen, and kidneys. In addition, no significant differences in histological features of the liver, kidney, and colon were observed between the NH and NC groups. These findings suggest the safety of WEH in C57BL/6J mice and indicate its potential as a source of EGT-rich functional ingredients for further health-related studies.

In behavioral experiments, WEH supplementation resulted in clearer locomotor trajectories in model mice, with movements primarily concentrated in the platform quadrant. The time spent in the target quadrant, the number of platform crossings, and the distance travelled all increased significantly. Liang et al. (12) also reported similar findings, assessing the effects of low, medium, and high doses of EGT-rich *Pleurotus eryngii* extract in an Aβ-induced AD mouse model, and found that all doses significantly reduced escape latency and escape distance, effectively alleviating memory deficits. Therefore, supplementation with EGT-rich WEH effectively reduced cognitive impairment in APP/PS1 mice. Furthermore, the high-dose AH group was significantly superior to the low-dose AL and ALE groups in terms of time spent in the target quadrant and travel distance, suggesting that higher concentrations of WEH had a greater beneficial effect on the model mice. Additionally, hippocampal apoptosis is a key contributor to cognitive dysfunction in AD, as neuronal damage in this region directly impairs learning and memory (20). In this study, supplementation with WEH significantly restored neuronal structural integrity in APP/PS1 mice, with hippocampal and cortical neuronal morphology approaching that of wild-type controls. A large prospective cohort study (Ohsaki Cohort 2006) including 13,230 older adults (≥65) in Japan reported that frequent mushroom consumption (≥3 times/week) was significantly associated with a lower risk of incident dementia (HR = 0.81, 95% CI: 0.69–0.95) (21). This finding suggests that regular mushroom consumption may help maintain cognitive function in the elderly. Additionally, *H. erinaceus* supplements improved recognition memory and reversed age-related cognitive decline in mice, boosting proliferating cell nuclear antigen and doublecortin expression in the hippocampus and cerebellum (22). These findings support our results, indicating that WEH supplementation effectively improved cognitive function in APP/PS1 mice.

Early AD is characterized by A*β* plaques, tau hyperphosphorylation, and glial cell activation, leading to neuroinflammation and cognitive decline (23). In this study, we found that WEH supplementation reduced hippocampal Aβ expression and improved tau pathology. Yang et al. (24) reported that EGT prevented Aβ-induced cognitive decline and significantly reduced Aβ accumulation in the hippocampus of Aβ1-40-treated mice. Whitmore et al. (25) found that EGT treatment modestly improved cognitive performance in 5XFAD mice, reducing Aβ plaque burden, oxidative stress, and impaired glucose metabolism. Li et al. (26) showed that EGT and lactoferrin together enhanced cognition and reduced Aβ accumulation and tau phosphorylation in APP/PS1 mice. These were similar to our results. Interestingly, ALE showed slightly higher Aβ levels than the WEH-treated group (AL and AH), suggesting that components other than EGT in *H. erinaceus* HE-17 may also contribute to the observed neuroprotective effects. Indeed, other bioactive compounds found in edible and medicinal fungi, such as polysaccharides, proteins, sterols, and terpenoids, are also considered safe and effective neuroprotective agents and promising therapeutic candidates for AD (27). These compounds generally exert beneficial effects through antioxidant and anti-inflammatory pathways, including the inhibition of oxidative stress, modulation of microglial activation, and suppression of amyloid-β accumulation. Compared with these compounds, EGT is a naturally occurring thiol derivative with a unique cellular uptake system mediated by the OCTN1 transporter, allowing its selective accumulation in tissues exposed to oxidative stress, including the brain (10). EGT may provide additional protection through its stability, targeted accumulation, and capacity to scavenge reactive oxygen and nitrogen species (24, 25). Despite these shared antioxidative and anti-inflammatory properties, differences in bioavailability, transport mechanisms, and metabolic stability may lead to distinct efficacy profiles among these compounds in AD models. Therefore, EGT-rich extracts such as WEH may represent a complementary dietary strategy alongside other bioactive compounds to maintain cognitive function and mitigate neurodegeneration. However, whether interactions among these components contribute to the enhanced effects of WEH remains to be investigated. Additionally, in the brain, glial cells, such as microglia and astrocytes, are essential for maintaining neuronal homeostasis (28). However, the accumulation of Aβ in AD pathogenesis elicits excessive glial responses, leading to chronic neuroinflammation and contributing to neurodegeneration (29). In early AD, activated microglia play a beneficial role by clearing Aβ via pinocytosis or phagocytosis (30, 31). Yet, as disease progresses, microglial function becomes impaired, diminishing their capacity to clear Aβ, which contributes to disease progression and cognitive decline (32, 33). In this study, WEH supplementation reduced IBA1 levels compared with the model control group, indicating that WEH can reduce microglial activation. Ishimoto et al. (34) found that EGT significantly inhibited intracellular reactive oxygen species (ROS) production and microglial activation, thereby inhibiting cellular hypertrophy. This was similar to our results, suggesting that WEH may attenuate AD pathology by reducing Aβ deposition and tau phosphorylation.

Neuroinflammation is increasingly recognized as an initiating factor in AD, promoting Aβ and p-tau accumulation (35). Within the innate immune response, NLRP3 inflammasomes, innate immune components expressed by macrophages and microglia, mediate caspase-1 activation and promote the secretion of pro-inflammatory cytokines within the CNS, thereby contributing to both systemic and neuroinflammatory responses (36, 37). Accumulating evidence indicates that the pro-inflammatory cytokines (IL-1β, IL-6, and TNF-*α*) are strongly associated with the progression of AD (38). In this study, supplementation with EGT-rich WEH significantly reduced pro-inflammatory cytokine levels (IL-1β, IL-6, and TNF-α) in APP/PS1 mice. It has been found that extracts from the white button mushroom exert protective effects during the progression of liver fibrosis by attenuating both inflammatory responses and oxidative stress (39). This was similar to our results, indicating that WEH exerts neuroprotective effects by suppressing neuroinflammation.

Oxidative stress plays a pivotal role in the development and progression of AD (3). Biomarkers such as T-AOC, SOD, CAT, and GSH are key components of the antioxidant defense system that scavenge ROS. In AD, decreased levels or activities of these antioxidants have been widely reported, indicating compromised cellular defense against oxidative stress (40–42). In this study, WEH supplementation markedly increased the activities of T-AOC, SOD, and CAT in APP/PS1 mice, whereas a notable elevation in GSH levels was observed exclusively in the AH group. One hypothesized mechanism suggests that Aβ may directly bind to antioxidant enzymes, thereby disrupting their normal activity and reducing their functional efficiency (43). EGT has been shown to inhibit Aβ accumulation in the hippocampus, reduce lipid peroxidation in neuronal cells, and enhance the activity of other endogenous antioxidants (44). Epidemiological evidence suggests that EGT-rich edible mushroom extracts may exert neuroprotective effects. The mechanism involves attenuating oxidative stress and neuroinflammation by reducing pro-inflammatory and oxidative markers, including IL-6, cyclooxygenase-2 (COX-2), and inducible nitric oxide synthase (NOS2), and enhancing antioxidant pathways, such as nuclear factor erythroid 2–related factor 2 (Nrf2) and SOD1. In addition, they improve synaptic function by upregulating glutamatergic receptors, thereby supporting hippocampal integrity (19). Liu et al. (45) reported that EGT contributes roughly 25% of the total antioxidant capacity observed in extracts of edible and medicinal mushrooms. Additionally, WEH supplementation significantly decreased MDA and NO levels and increased Ach levels, with greater effects observed at the high dose. D-galactose induces damage to hippocampal neurons by increasing ROS production and advanced glycation end product accumulation, leading to oxidative stress, mitochondrial dysfunction, and neuroinflammation. EGT mitigates these effects by activating the Nrf2/HO-1 pathway, reducing MDA levels, and enhancing T-SOD activity. Furthermore, EGT improves mitochondrial function via the AMPK/SIRT1/PGC-1α pathway and suppresses neuroinflammatory responses, thereby protecting hippocampal neurons from D-galactose-induced damage (46). Ryu et al. (47) found that golden oyster mushroom extract exerts neuroprotective effects by preventing scopolamine-induced damage to the cholinergic system and disruption of the antioxidant defense system. These findings were similar to our results and suggest that WEH may preserve neuronal function and exert neuroprotective effects by modulating oxidative stress pathways in the brain.

Dysbiosis of the gut microbiota has been strongly associated with AD. The gut–brain axis mediates bidirectional communication between the gut and the CNS, and its disruption can alter microbial metabolite production, promote neuroinflammation, and impair barrier integrity, thereby contributing to AD progression (48). A study reported that individuals with AD exhibit reduced microbial diversity and an increased abundance of *Proteobacteria* (49), and elevated levels of *Fusobacteriota* have been suggested as potential biomarkers for AD (50). A similar trend was observed in the present study. Notably, low-dose WEH significantly reduced the relative abundance of *Fusobacteriota* and *Proteobacteria*, two taxa commonly associated with pro-inflammatory states and gut microbiota dysbiosis. In addition, WEH supplementation markedly increased the overall abundance of gut microbiota at the phylum level in APP/PS1 mice. At the genus level, WEH supplementation also increased the relative abundance of *Lactobacillus*. Studies have demonstrated that *Lactobacillus* can enhance acetylcholine levels, thereby improving cognitive function and spatial memory (51–53). Additionally, *Bifidobacterium breve* HNXY26M4 has been reported to ameliorate cognitive deficits, reduce neuroinflammation and synaptic dysfunction, restore gut microbiota composition and SCFAs levels, and strengthen gut barrier integrity in APP/PS1 mice (54). These findings are similar to our results, suggesting that WEH exerts neuroprotective effects, at least in part, by modulating gut microbiota composition and promoting *Lactobacillus*, which may contribute to improved cognitive function and reduced AD-related pathology. Notably, although high-dose WEH showed more pronounced neuroprotective effects in certain parameters, the gut microbiota alterations did not exhibit a clear dose–response relationship. This inconsistency may reflect the inherent complexity, inter-individual variability, and dynamic nature of the gut microbial ecosystem.

## 5 Conclusion

In conclusion, WEH and pure EGT alleviated Aβ deposition and tau phosphorylation in APP/PS1 mice, while also reducing oxidative stress and neuroinflammation. High-dose WEH showed relatively stronger neuroprotective effects. Low-dose WEH was associated with improvements in gut microbiota composition and diversity, characterized by a reduced abundance of *Fusobacteriota* and *Proteobacteria*, and an increased abundance of *Lactobacillus*. These results suggest that WEH, likely through synergistic interactions among EGT and other constituents, may represent a potential dietary intervention for AD by modulating oxidative stress, inflammation, and the gut microbiota. However, this study only included male mice, which may limit the generalizability of the findings. Given the potential sex differences in AD, further studies in female animals are needed.

## Data Availability

The original contributions presented in the study are publicly available. This data can be found here: NCBI Sequence Read Archive (SRA), BioProject accession number PRJNA1463100.

## Footnotes

1
https://cloud.oebiotech.com
