# Supplementary material for: Ergothioneine-rich water extracts of Hericium erinaceus HE-17 alleviate Alzheimer’s disease in mice by regulating oxidative stress, inflammation, and the gut microenvironment
Source: Front Nutr. 2026 May 21;13:1835714. doi: 10.3389/fnut.2026.1835714 (PMC13233374; doi:10.3389/fnut.2026.1835714)
Supplement: Supplementary file 1 [file Table_1.docx]

Supplementary Material

**Supplementary Table 1.** Factor and level table for orthogonal test of culture conditions.

|  | Factor | | |
| --- | --- | --- | --- |
|  | A  Fermentation time (d) | B  Water content (%) | C |
| Level |  |  | Substrate loading (g) |
| 1 | 6 | 60 | 10 |
| 2 | 9 | 75 | 25 |
| 3 | 12 | 90 | 40 |

**Supplementary Table 2.** Data of orthogonal test for culture conditions.

|  | 1 | 2 | 3 | 4 |  |
| --- | --- | --- | --- | --- | --- |
| Factor | Fermentation time | Water content | Substrate loading |  | EGT content |
| Experiment 1 | 1 | 1 | 1 | 1 | 0.59331 |
| Experiment 2 | 1 | 2 | 2 | 2 | 0.38548 |
| Experiment 3 | 1 | 3 | 3 | 3 | 0.30292 |
| Experiment 4 | 2 | 1 | 2 | 3 | 0.42629 |
| Experiment 5 | 2 | 2 | 3 | 1 | 0.3533 |
| Experiment 6 | 2 | 3 | 1 | 2 | 0.43483 |
| Experiment 7 | 3 | 1 | 3 | 2 | 0.39592 |
| Experiment 8 | 3 | 2 | 1 | 3 | 0.5041 |
| Experiment 9 | 3 | 3 | 2 | 1 | 0.3646 |
| Mean 1 | 0.427 | 0.472 | 0.511 | 0.437 |  |
| Mean 2 | 0.405 | 0.414 | 0.392 | 0.405 |  |
| Mean 3 | 0.422 | 0.367 | 0.351 | 0.411 |  |
| Range | 0.022 | 0.105 | 0.160 | 0.032 |  |

**Supplementary Table 3.** Orthogonal factor and level table of amino acids.

|  | Factor | | |
| --- | --- | --- | --- |
|  | A | B | C |
| Level | Met g/20 g | His g/20 g | Cys g/20 g |
| 1 | 0.05 | 0 | 0.01 |
| 2 | 0.10 | 0.05 | 0.03 |
| 3 | 0.15 | 0.1 | 0.04 |
| 4 | 0.20 | 0.15 | 0.05 |

**Supplementary Table 4.** Data of orthogonal experiment on amino acids.

|  | 1 | 2 | 3 | 4 | 5 |  |
| --- | --- | --- | --- | --- | --- | --- |
| Factor | Met | His | Cys |  |  | EGT content |
| Experiment 1 | 1 | 1 | 1 | 1 | 1 | 0.657 |
| Experiment 2 | 1 | 2 | 2 | 2 | 2 | 0.55787 |
| Experiment 3 | 1 | 3 | 3 | 3 | 3 | 0.67954 |
| Experiment 4 | 1 | 4 | 4 | 4 | 4 | 0.56497 |
| Experiment 5 | 2 | 1 | 2 | 3 | 4 | 0.6392 |
| Experiment 6 | 2 | 2 | 1 | 4 | 3 | 0.68157 |
| Experiment 7 | 2 | 3 | 4 | 1 | 2 | 0.81034 |
| Experiment 8 | 2 | 4 | 3 | 2 | 1 | 0.70489 |
| Experiment 9 | 3 | 1 | 3 | 4 | 2 | 0.66028 |
| Experiment 10 | 3 | 2 | 4 | 3 | 1 | 0.73835 |
| Experiment 11 | 3 | 3 | 1 | 2 | 4 | 0.86915 |
| Experiment 12 | 3 | 4 | 2 | 1 | 3 | 0.67143 |
| Experiment 13 | 4 | 1 | 4 | 2 | 3 | 0.77992 |
| Experiment 14 | 4 | 2 | 3 | 1 | 4 | 0.88537 |
| Experiment 15 | 4 | 3 | 2 | 4 | 1 | 0.747 |
| Experiment 16 | 4 | 4 | 1 | 3 | 2 | 0.98981 |
| Mean 1 | 0.615 | 0.684 | 0.799 | 0.756 | 0.712 |  |
| Mean 2 | 0.709 | 0.716 | 0.654 | 0.728 | 0.755 |  |
| Mean 3 | 0.735 | 0.777 | 0.733 | 0.762 | 0.703 |  |
| Mean 4 | 0.851 | 0.733 | 0.723 | 0.663 | 0.740 |  |
| Range | 0.236 | 0.093 | 0.145 | 0.099 | 0.052 |  |

**Supplementary Table 5.** Alpha Diversity Analysis (n=6). ACE, Abundance-based Coverage Estimator metric. Chao1, Chao1 richness estimator. Simpson, Simpson's diversity index. Shannon, Shannon-Wiener diversity index. WEH, water extract of *Hericium erinaceus* HE-17; NC, wild-type control group; NH, wild-type mice treated with high-dose WEH (2 g/kg BW/d); AC, APP/PS1 model control group; AL, APP/PS1 mice treated with low-dose WEH (0.50 g/kg BW/d); AH, APP/PS1 mice treated with high-dose WEH (2 g/kg BW/d); ALE, APP/PS1 mice treated with ergothioneine (1.44 mg/kg BW/d).

| Group | ACE | Chao 1 | Simpson | Shannon |
| --- | --- | --- | --- | --- |
| NC | 296.23+65.70b | 296.82+64.40b | 0.97+0.01b | 6.37+0.46a |
| NH | 356.44+38.44a | 355.39+37.27a | 0.98+0.00ab | 6.70+0.14a |
| AC | 327.44+52.07ab | 327.53+53.40ab | 0.97+0.00ab | 6.59+0.25a |
| AL | 321.56+23.94ab | 322.17+24.49ab | 0.97+0.00ab | 6.53+0.19a |
| ALE | 321.32+28.02ab | 320.71+28.17ab | 0.98+0.00a | 6.64+0.17a |
| AH | 344.58+39.92ab | 343.49+39.86ab | 0.97+0.00ab | 6.60+0.23a |

**Supplementary Table 6.** Anosim analysis.

| Method name | ANOSIM |
| --- | --- |
| Test statistic name | R |
| Sample size | 36 |
| Number of groups | 6 |
| Test statistic | 0.519 |
| p-value | 0.001 |
| Number of permutations | 999 |


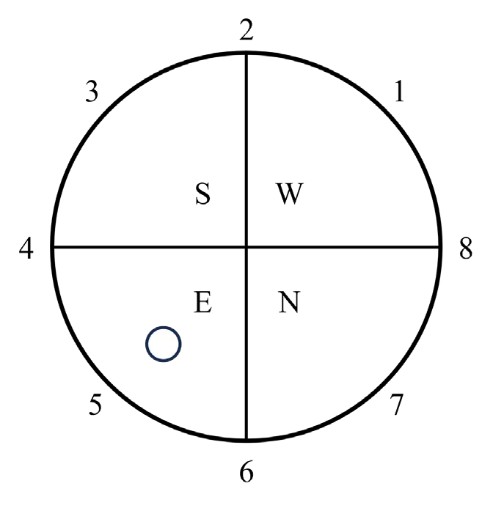


**Supplementary Figure 1.** Distribution of Morris Water Maze quadrants and water entry points of mice.


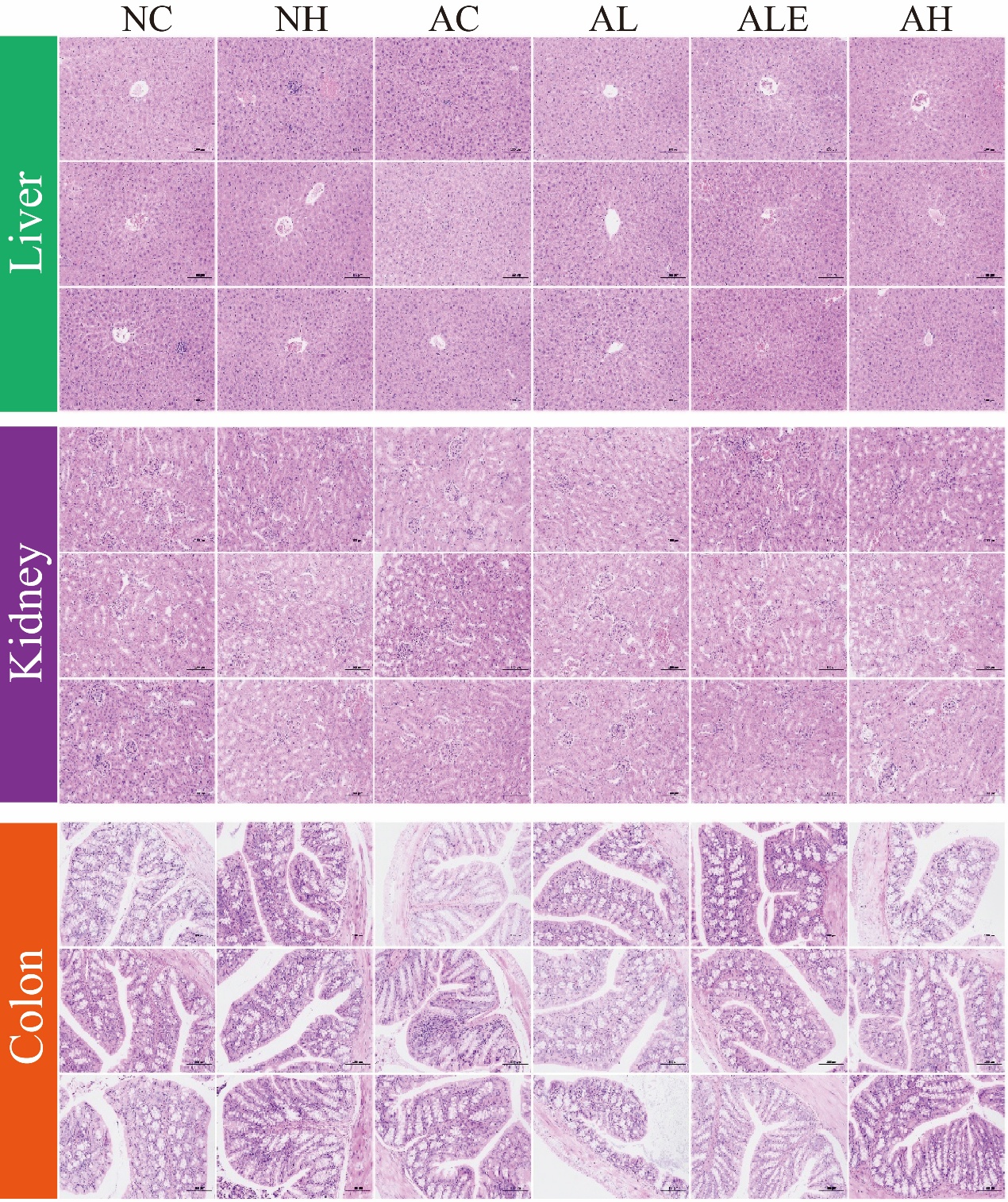


**Supplementary Figure 2.** H&E staining image of liver lobe tips, kidney pea-shaped sections and colon loop sections in C57BL/6J mice. WEH, water extract of *Hericium erinaceus* HE-17; NC, wild-type control group; NH, wild-type mice treated with high-dose WEH (2 g/kg BW/d); AC, APP/PS1 model control group; AL, APP/PS1 mice treated with low-dose WEH (0.50 g/kg BW/d); AH, APP/PS1 mice treated with high-dose WEH (2 g/kg BW/d); ALE, APP/PS1 mice treated with ergothioneine (1.44 mg/kg BW/d).


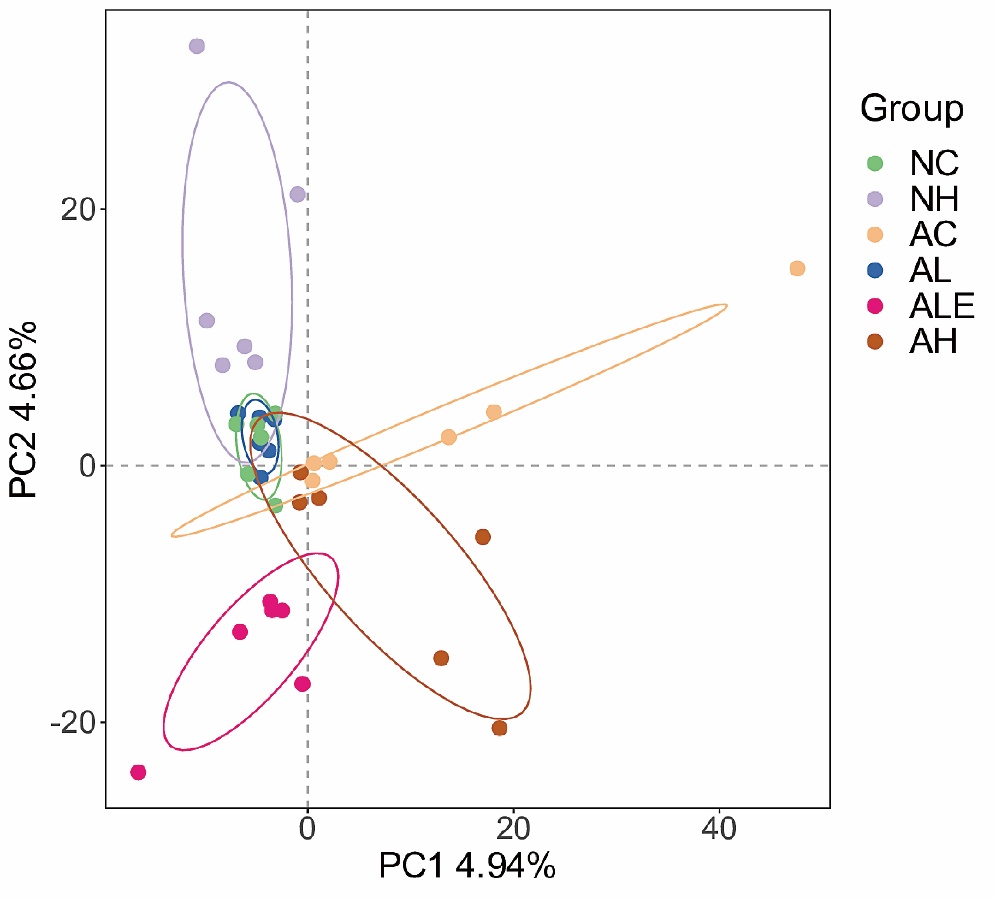


**Supplementary Figure 3.** PCoA analysis of fecal microbiota in mice. WEH, water extract of *Hericium erinaceus* HE-17; NC, wild-type control group; NH, wild-type mice treated with high-dose WEH (2 g/kg BW/d); AC, APP/PS1 model control group; AL, APP/PS1 mice treated with low-dose WEH (0.50 g/kg BW/d); AH, APP/PS1 mice treated with high-dose WEH (2 g/kg BW/d); ALE, APP/PS1 mice treated with ergothioneine (1.44 mg/kg BW/d).
